# The oestrogen receptor alpha-regulated lncRNA NEAT1 is a critical modulator of prostate cancer

**DOI:** 10.1038/ncomms6383

**Published:** 2014-11-21

**Authors:** Dimple Chakravarty, Andrea Sboner, Sujit S. Nair, Eugenia Giannopoulou, Ruohan Li, Sven Hennig, Juan Miguel Mosquera, Jonathan Pauwels, Kyung Park, Myriam Kossai, Theresa Y. MacDonald, Jacqueline Fontugne, Nicholas Erho, Ismael A. Vergara, Mercedeh Ghadessi, Elai Davicioni, Robert B. Jenkins, Nallasivam Palanisamy, Zhengming Chen, Shinichi Nakagawa, Tetsuro Hirose, Neil H. Bander, Himisha Beltran, Archa H. Fox, Olivier Elemento, Mark A. Rubin

**Affiliations:** 1 Department of Pathology and Laboratory Medicine, Weill Medical College of Cornell University, 413 East 69th Street, Room 1402, New York, New York 10021, USA; 2 Institute for Precision Medicine, Weill Medical College of Cornell University and New York Presbyterian Hospital, New York, New York 10021, USA; 3 Institute for Computational Biomedicine, Weill Cornell Medical College of Cornell University, New York, New York 10021, USA; 4 Department of Biochemistry and Molecular Medicine, School of Medicine and Health Sciences, George Washington University, Washington DC 20037, USA; 5 Biological Sciences Department, New York City College of Technology, City University of New York, Brooklyn, New York 11201, USA; 6 Arthritis and Tissue Degeneration Program and the David Z. Rosensweig Genomics Research Center, Hospital for Special Surgery, New York, New York 10021, USA; 7 School of Biomedical, Biomolecular and Chemical Sciences, University of Western Australia, Crawley, Western Australia 6009, Australia; 8 Chemical Genomics Centre, 44227 Dortmund, Germany; 9 Research and Development, GenomeDx Biosciences, Vancouver, British Columbia, Canada V6B 1B8; 10 Department of Pathology and Laboratory Medicine, Mayo Clinic, Rochester, Minnesota 55905, USA; 11 Michigan Center for Translational Pathology, University of Michigan, Ann Arbor, Michigan 48105, USA; 12 Henry Ford Health System, Medical Group Urology - Prostate Cancer Research, 1 Ford Place, Room 2D26, Detroit, Michigan 48202, USA; 13 Division of Biostatistics and Epidemiology, Department of Public Health, Weill Cornell Medical College, New York, New York 10021, USA; 14 RNA Biology Laboratory, RIKEN Advanced Research Institute, Hirosawa 2-1, Wako 351-0198, Japan; 15 Institute for Genetic Medicine, Hokkaido University, Kita-15 Nishi-7, Kita-ku, Sapporo 060-0815, Japan; 16 Department of Urology, Weill Cornell Medical College of Cornell University, New York, New York 10021, USA

## Abstract

The androgen receptor (AR) plays a central role in establishing an oncogenic cascade that drives prostate cancer progression. Some prostate cancers escape androgen dependence and are often associated with an aggressive phenotype. The oestrogen receptor alpha (ERα) is expressed in prostate cancers, independent of AR status. However, the role of ERα remains elusive. Using a combination of chromatin immunoprecipitation (ChIP) and RNA-sequencing data, we identified an ERα-specific non-coding transcriptome signature. Among putatively ERα-regulated intergenic long non-coding RNAs (lncRNAs), we identified nuclear enriched abundant transcript 1 (NEAT1) as the most significantly overexpressed lncRNA in prostate cancer. Analysis of two large clinical cohorts also revealed that NEAT1 expression is associated with prostate cancer progression. Prostate cancer cells expressing high levels of NEAT1 were recalcitrant to androgen or AR antagonists. Finally, we provide evidence that NEAT1 drives oncogenic growth by altering the epigenetic landscape of target gene promoters to favour transcription.

## Abstract (web summary)

While prostate cancer predominantly exhibits androgen dependence, oestrogen receptor (ER) signalling is also involved. Here, Chakravarty *et al.* show that ERα regulates the expression of the NEAT1 long non-coding RNA, which in turn promotes tumorigenesis by maintaining an oncogenic programme/cascade.

## Graphical abstract

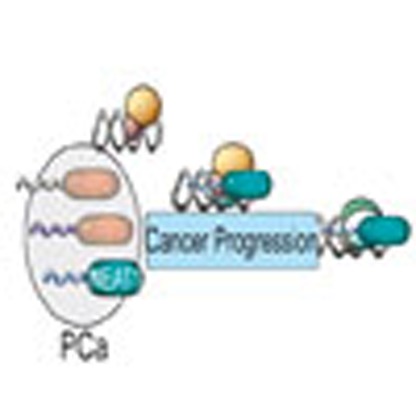

Steroid receptors are key transducers of hormone signalling and control a wide spectrum of tissue-specific functions that are critical for the physiological homeostasis of reproductive organs. Aberrant or deregulated expressions of steroid nuclear receptors are often associated with cancer progression and have been a major target for therapeutic intervention. The androgen receptor (AR) plays a central role in the progression of prostate cancer^1^. Androgen ablation is highly effective in treating metastatic prostate cancer, although resistance inevitably develops leading to castrate-resistant prostate cancer (CRPC). Most cases of CRPC remain dependent on AR signalling, which has led to the clinical development and recent approval of potent AR-targeted therapies for CRPC (that is, abiraterone and enzalutamide)^2^,^3^. However, similar to first-generation anti-androgen therapies, patients develop resistance to these second-generation hormonal therapies. How CRPC tumours bypass AR signalling is emerging as a significant area of investigation. Many view co-targeting therapies as an important next step to managing the inevitable emergence of resistance to single-agent treatments, but critical to co-targeting is the identification of other biological pathways that drive disease progression and the development of strategies that can target judgmental pathways.

In CRPC, cross-talk between oestrogen- and androgen-signalling pathways may present an opportunity for clinical intervention. Oestrogen receptor (ER) signalling through ERα increases with prostate cancer progression^4^,^5^,^6^ and can drive important oncogenic events, including TMPRSS2-ERG expression^7^. Although ERα signalling has been extensively studied in breast cancer^8^,^9^,^10^, our understanding of the potential impact of this nuclear receptor on prostate physiology is less clear. Nevertheless, the connection is a particularly intriguing concept given that most cases of prostate cancer arise in the sixth decade of life, a time when testosterone levels are decreasing and oestrogens are increasing in men. Mouse models suggest that antagonism of ERα may diminish prostate carcinogenesis^4^.

We posit that ERα is an important alternate signalling pathway for the transcriptional regulation of prostate cancer, allowing refractory disease to bypass androgen/AR signalling. Herein, we provide experimental evidence to support this hypothesis and demonstrate a functional specialization and distinct genomic role of this nuclear receptor in prostate cancer, with significant implications for prognosis and management. We show that ERα is recruited to both coding and non-coding regions of the prostate genome and orchestrates expression of non-coding regulatory RNAs.

We identified nuclear enriched abundant transcript 1 (NEAT1) long non-coding RNA (lncRNA) as a potential target of ERα and as an important mediator for maintenance of prostate cancer. NEAT1 functions as a transcriptional regulator and contributes to a cancer-favourable transcriptome, thereby promoting tumorigenesis in experimental animal models. Our analysis of the transcriptional role of NEAT1 identified functions beyond its previously characterized role in maintaining the integrity of subnuclear organelles called paraspeckles^5^. We demonstrate that NEAT1 is recruited to the chromatin of well-characterized prostate cancer genes and contributes to an epigenetic ‘on’ state. Analysis of two large clinical cohorts nominated NEAT1 as a novel biomarker of disease progression. Given its significance within the ERα signalling pathway, we propose that targeting NEAT1 might represent a novel and important therapeutic strategy for the treatment of prostate cancer.

## Results

### ERα in transcriptional regulation of prostate cancer

**Figure 1:**
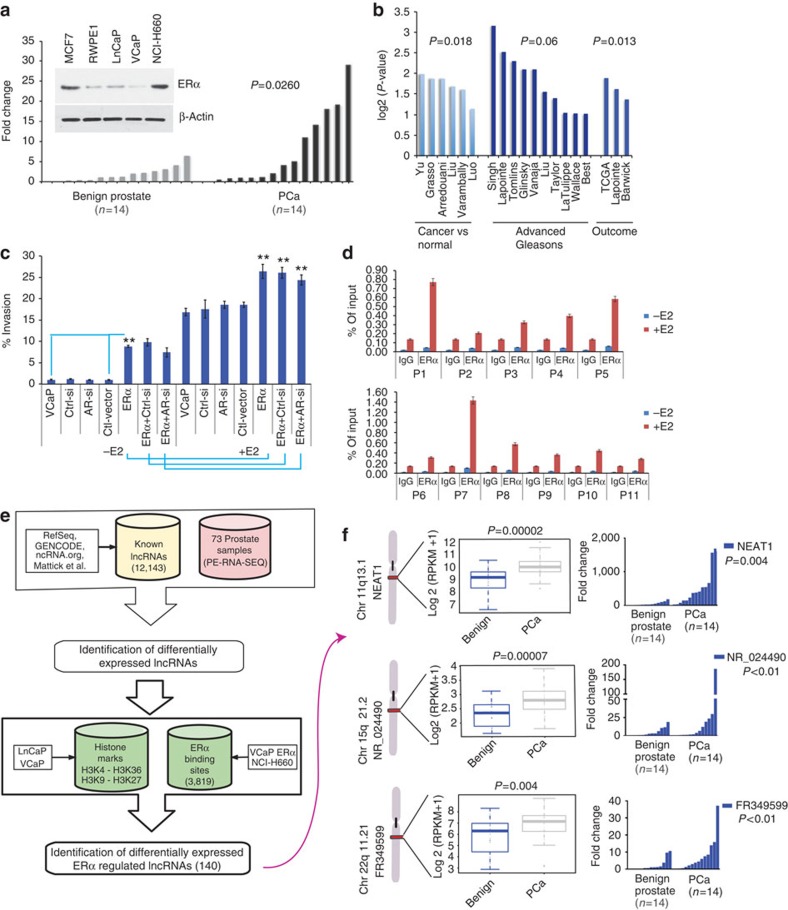
ERα plays a distinct role in prostate cancer. (**a**) ERα is upregulated in prostate cancer compared with matched benign controls. Waterfall plots depict the qRT-PCR expression levels of ERα mRNA in an independent cohort of benign (*n*=14) and PCa (*n*=14). (inset A) The expression of ERα in different prostate cancer cell lines was determined by western blotting and compared with MCF7, a breast cancer cell line. (**b**) Analysis of ERα expression in Oncomine public data sets of normal versus prostate cancer and advanced disease. (**c**) Invasion of VCaP and VCaP ERα cells analysed 48 h post treatment with vehicle control or E2 (10 nM) in the presence of control or AR-siRNA. Results are expressed as the mean±s.d. of three independent experiments. Student’s *t*-test was performed for comparisons (% Invasion) between −E2 and +E2 conditions for ERα, ERα-Ctrl siRNA and AR-siRNA, and **P<0.01 were considered statistically significant. (**d**) Recruitment of endogenous ERα to target gene chromatin was analysed in VCaP cells with or without E2 treatment. Results are expressed as the percentage of input of two independent experiments. Error bars represent the range of data. (**e**) Computational pipeline for identification of ERα-regulated lncRNAs upregulated in prostate cancer: a schematic overview of the methodology employed to identify ERα-regulated lncRNAs that are differentially expressed between benign versus prostate cancer and prostate cancer versus NEPC. (**f**) Box plots show expression levels of the top three ERα-regulated lncRNAs from 26 benign and 40 PCa cases, with ideogram depicting their chromosomal position. Waterfall plots depict the qRT-PCR expression levels on an independent cohort of benign (*n*=14) and PCa (*n*=14) of the three nominated lncRNAs: NEAT1, NR_024490 and FR349599.

To elucidate the role of ERα in prostate cancer, we analysed ERα protein and transcript levels in a panel of prostate cancer cell lines (*n*=5) and in a cohort of matched benign prostate tissue (*n*=14) and prostate adenocarcinoma (PCa) (*n*=14), respectively. We observed that ERα was significantly upregulated (*P*=0.03) in prostate tumours compared with benign tissues (Fig. 1a). To determine the clinical relevance of ERα in prostate cancer, we performed immunohistochemistry using a tissue microarray composed of tissue cores from 64 samples of benign prostate tissue, 16 high-grade prostate intraepithelial neoplasia, 292 PCa, and 42 neuroendocrine prostate cancer (NEPC). Representative photomicrographs are depicted in Supplementary Fig. 1a. Although benign prostate had only low expression levels of ERα, ERα was detected in adenocarcinoma and the adjacent high-grade prostate intraepithelial neoplasia through focal nuclear and cytoplasmic staining (Supplementary Fig. 1a). ERα is overexpressed in a significant number of prostate cancer cohorts. It was also found to be overexpressed in prostate cancers with high Gleason score (GS) compared with those with low GS as well as in those with tumour recurrence when analysed via the Oncomine^6^ database (Fig. 1b)^7^,^8^,^9^,^10^,^11^,^12^,^13^,^14^,^15^,^16^,^17^,^18^,^19^,^20^,^21^,^22^. Analysis of subcellular distribution in prostate cancer cell lines revealed significant nuclear distribution of ERα in all cell lines tested (Supplementary Fig. 1b). ERα protein levels were similar in both AR-positive LnCaP and VCaP cells (Fig. 1a, inset). We used parental VCaP and the ERα-positive prostate cancer cell line NCI-H660 as model cell lines to further explore and delineate the specific contribution of ERα to prostate cancer. A ligand-dependent modulation of invasive potential was observed in VCaP cells on oestrogen (E2) treatment (Fig. 1c). These results suggest that a functionally relevant, ligand-dependent ERα signalling pathway is active in prostate cancer cell lines.

To further understand the impact of ERα, we generated VCaP cells that overexpress ERα (VCaP ERα). Stable expression of ERα was confirmed by western blotting (Supplementary Fig. 1c). VCaP ERα exhibited significantly higher invasive potential than VCaP parental cells or the vector control cells (Fig. 1c). Intriguingly, the noted effects of ERα overexpression were independent of AR status, as experimental silencing of AR in VCaP ERα cells (Supplementary Fig. 1c) did not compromise the increased invasive potential of E2-treated VCaP ERα cells (Fig. 1c). These data suggest that prostate cancer cells can use alternate nuclear receptor signalling (for example, ERα signalling) to propagate, and understanding these mechanisms will help discern the complete spectrum of key regulators of prostate cancer progression.

Studies have established ERα’s dominant role in transcriptional regulation of target genes in breast cancer^23^,^24^. Likewise, high nuclear levels of ERα in prostate cancer cells (Supplementary Fig. 1b) and their direct association with chromatin implicate ERα in the transcriptional regulation of this cancer, as well. We used ERα chromatin immunoprecipitation coupled with high-throughput sequencing (ChIP-seq) in VCaP cells, with and without E2 treatment, and also in VCaP ERα and NCI-H660 cells with E2 treatment to investigate the underlying mechanisms by which ERα might drive a transcriptional programme in prostate cancer. The majority of ERα-binding sites were cell specific (Supplementary Fig. 1d). Analysis of the ChIP-seq data for ERα in NCI-H660 and VCaP ERα cells revealed that 64.9% of ERα binding occurred within intergenic regions of the prostate genome. This fraction is higher than the expected fraction if peaks were randomly distributed across the genome (*P*=3e−05) (Supplementary Fig. 1e).

Using publicly available data sets^25^, we found that 28% of the intergenic ERα-binding sites in the prostate cancer genome (from VCaP ERα and NCI-H660 cell lines) overlapped with the active histone marks trimethylated lysine 4 of histone H3 (H3K4me3) and trimethylated lysine 36 of histone H3 (H3K36me3) (*P*<1e−7). On the other hand, 20.7% of those sites overlapped with histone marks typical of inactive chromatin, such as trimethylated lysine 9 of histone H3 or trimethylated lysine 27 of histone H3. To prioritize experimental validation of ERα targets, we ranked the peaks according to the average *P*-value determined by the peak-calling algorithm ChIPSeeqer^26^ and selected the highest ranking peaks for further analysis. We analysed recruitment of endogenous ERα to the top 11 binding sites in parental VCaP cells (Fig. 1d), providing an experimental validation of the ChIP-seq data. A significantly higher recruitment of ERα was evident at the binding sites compared with control IgG.

Given the enhanced recruitment of ERα to intergenic regions of the prostate genome, we evaluated the likelihood that ERα might influence transcriptional output and thereby the repertoire of non-coding RNA in the context of prostate cancer. We thus analysed the abundance of non-coding transcripts in RNA-seq data derived from a cohort of 73 prostate tissues, which included 26 benign prostate samples, 40 PCa and 7 NEPC (Supplementary Dataset 1), focusing our analysis on 6,850 intergenic lncRNAs out of 12,143 known lncRNAs (see Supplementary Methods). We identified 1,314 and 1,399 intergenic lncRNAs that are differentially expressed between benign and PCa, and between PCa and NEPC, respectively (false discovery rate <0.01). We identified 140 intergenic lncRNAs putatively regulated by ERα (Fig. 1e and Supplementary Dataset 2). An analysis of AR-binding sites^25^ identified 98 lncRNAs that have an AR-binding site within the promoter. This supported the view that ERα might significantly influence the non-coding transcriptome in prostate cancer. Using the RNA-seq data on VCaP and VCaP ERα cell lines to validate the expression levels of the top differentially expressed ERα-regulated lncRNAs, we selected six potential candidate lncRNAs that had higher expression in VCaP ERα compared with VCaP. We used quantitative real-time PCR (qRT–PCR) to validate expression for these six ERα-regulated lncRNAs in VCaP and VCaP ERα-expressing cell lines (Supplementary Fig. 1f). Expression of three of these lncRNAs was further determined in a cohort of 28 matched benign and prostate cancer samples, confirming upregulation of these three nominated lncRNAs in prostate cancer compared with benign prostate (Fig. 1f). Taken together, these analyses indicate that ERα is a transcriptional regulator of the non-coding transcriptome in prostate cancer.

Among the putatively ERα-regulated intergenic lncRNAs, we identified NEAT1 as the most significantly overexpressed lncRNA in prostate cancer versus benign prostate in our patient cohort (73 samples) (Fig. 1f and Supplementary Dataset 2). The *NEAT1* gene is located on chromosome 11q13.1 and produces two RNA isoforms that overlap completely at the 5′-end. The shorter isoform (hereafter abbreviated as NEAT1/NEAT1_1) is 3.7 kB in length and more abundant than the longer, 23 kB isoform (NEAT1_2)^27^. NEAT1 lncRNA is essential for the formation of subnuclear bodies called paraspeckles^27^, and although both isoforms localize to paraspeckles, their physiological role in prostate cancer remains unknown.

### ERα-regulated NEAT1 lncRNA is upregulated in prostate cancer

**Figure 2:**
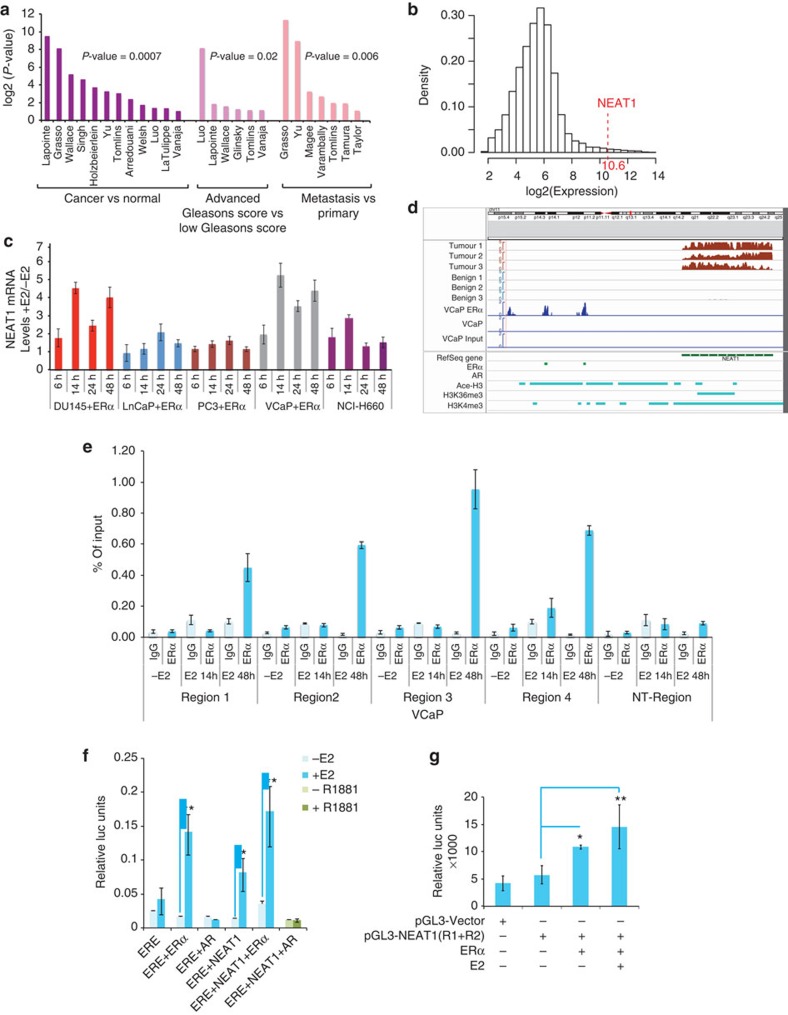
ERα regulated NEAT1 lncRNA is upregulated in prostate cancer. (**a**) NEAT1 is overexpressed in various prostate data sets (Oncomine). (**b**) Distribution of the median expression of all genes (core transcript clusters) on the Human Exon 1.0 ST array in the pooled Mayo Clinic cohort (*n*=594). NEAT1’s expression ranks in the 99th percentile of all genes on the array. (**c**) Expression of NEAT1 with/without ERα overexpression and E2 treatment (10 nM) at different time points in a panel of prostate cancer cell lines. Results are expressed as the mean±s.d. of three independent experiments. (**d**) View of NEAT1 genomic location indicates presence of two ERα-binding sites in the promoter region. Read coverage tracks derived from RNA-sequencing data indicates a higher abundance of NEAT1 transcripts in PCa compared with benign tumours in three representative cases. The figure also reports the ChIP-sequencing coverage tracks for ERα (VCaP ERα, VCaP and input DNA as control). The bottom panel shows the binding sites of ERα, AR (GEO Accession GSM353651-tissue AR (ref. 25)), Ace-H3, H3K4me3 and H3K36me3 in VCaP cell line (GEO Accession GSM353629, GSM353620 and GSM353624 (ref. 25)), respectively. (**e**) Chromatin immunoprecipitation followed by qPCR to study ERα recruitment to NEAT1 promoter in VCaP cells with/without E2 treatment (10 nM) was performed with primers spanning the binding regions identified by ERα ChIP-seq data. Primers for nonspecific region were used as negative control for ChIP studies. Results are expressed as percentage of input from two independent experiments. Vertical error bars represent the range of data. (**f**) Luciferase-based promoter reporter assays was used to analyse effect of ERα and/or AR on ERE-Luc promoter in VCaP cells. Cells were transiently transfected with the (ERE)3-SV40-luc reporter plasmid and ERα, or AR-treated with/without E2 or R1881 (1 nM) for 48 h. Results are expressed as the mean±s.d calculated from three independent experiments. (**g**) Luciferase-based promoter reporter assays were used to analyse NEAT1 promoter activity following ERα expression −/+E2 (10 nM) for 24 h. Results are expressed as the means±s.d. calculated from three independent experiments. Student’s *t*-test was performed for comparisons where indicated, and *P<0.05 and **P<0.01 were considered statistically significant.

In the Oncomine database, we observed significant overexpression of NEAT1 lncRNA in several prostate cancer data sets (normal versus cancer) and aggressive prostate cancer (Fig. 2a)^7^,^10^,^11^,^12^,^13^,^14^,^15^,^16^,^17^,^18^,^19^,^20^,^21^,^22^,^28^,^29^,^30^,^31^. We first confirmed that amplification of chromosome 11q (where NEAT1 resides) was not seen across 109 adenocarcinoma cases^32^, eliminating chromosome 11q13.1 amplification as an explanation for high NEAT1 expression (Supplementary Fig. 2a)^33^,^34^. The expression of NEAT1 in two radical prostatectomy cohorts with long-term clinical follow-up from the Mayo Clinic^35^,^36^ was measured using Affymetrix HuEx microarrays (see Methods). Supplementary Table 1 contains the patient characteristics of the data sets. NEAT1’s expression ranked in the 99th percentile of all genes on the microarray (Fig. 2b). We determined levels of NEAT1 by RNA *in situ* hybridization (ISH) in a tissue microarray that included 16 benign prostate tissues, 21 PCa, 12 PCa with neuroendocrine differentiation and 7 NEPC cases. NEAT1 was found to be highly expressed in prostate cancer compared with that in benign tissue (Supplementary Fig. 2b).

We observed that in a panel of prostate cancer cell lines, ERα overexpression and E2 treatment upregulated NEAT1 transcript levels in a time-dependent manner (Fig. 2c). In DU145, an ERG-negative cell line^37^, E2/ERα signalling was intact (Fig. 2c), supporting an ERG-independent phenomenon. Following ERα overexpression, we also recorded an increase in expression of the long isoform NEAT1_2 (Supplementary Fig. 2c). This was not surprising as both isoforms of NEAT1 are driven by the same promoter^38^. The preferential upregulation and increase in the NEAT1 long form alone is not well understood and is not further addressed in this study. Interestingly, knockdown of ERβ did not alter NEAT1 levels, suggesting that NEAT1 regulation is specific for ERα (Supplementary Fig. 2d).

NEAT1 was originally identified localized to subnuclear organelles called paraspeckles that are free of chromatin and function as repositories of edited RNA and a number of nuclear RNA-binding proteins^5^. Loss of NEAT1 dramatically reduces the formation of paraspeckles. Treatment of VCaP cells with E2 resulted in re-distribution of NEAT1 from paraspeckles to an enhanced distribution throughout the nucleus (Supplementary Fig. 2e).

We inspected our ERα ChIP-seq data in VCaP ERα and NCI-H660 cells, and identified two ERα-binding sites on the NEAT1 promoter (Fig. 2d). Analysis of chromatin marks using ChIP-seq data sets for histone marks^25^ revealed the presence of active histone marks H3 Acetyl K9 and H3K4me3 in the promoter region of NEAT1, while H3K36Me3 marks were abundant in the gene body (Fig. 2d). A recent study revealed that bivalent H3K4Me3 and H3K36Me3 marks are indicators of functional transcriptional loci from the non-coding genome^39^. ERα recruitment to specific regions of the NEAT1 promoter was independently validated by ERα ChIP in VCaP, VCaP ERα and NCI-H660 cells (Fig. 2e and Supplementary Fig. 2f,g) using specific primers encompassing ERα-binding sites in the NEAT1 promoter. We found that a functional oestrogen/ERα signalling pathway was active in VCaP cells, as determined by reporter-based estrogen response element (ERE) luciferase assays in VCaP cells, with ERα and AR overexpression, and E2 or R1881 treatment, respectively, for 48 h (Fig. 2f). To further test whether ERα is required for NEAT1 transcriptional activation, we generated luciferase promoter reporter constructs with both ERα-binding sites upstream of the luciferase-coding region. Luciferase reporter assays in VCaP cells confirmed that NEAT1 promoter activity was upregulated in an ERα-dependent manner and further enhanced with E2 treatment (Fig. 2g).

### ERα and NEAT1 regulate several prostate cancer genes

**Figure 3:**
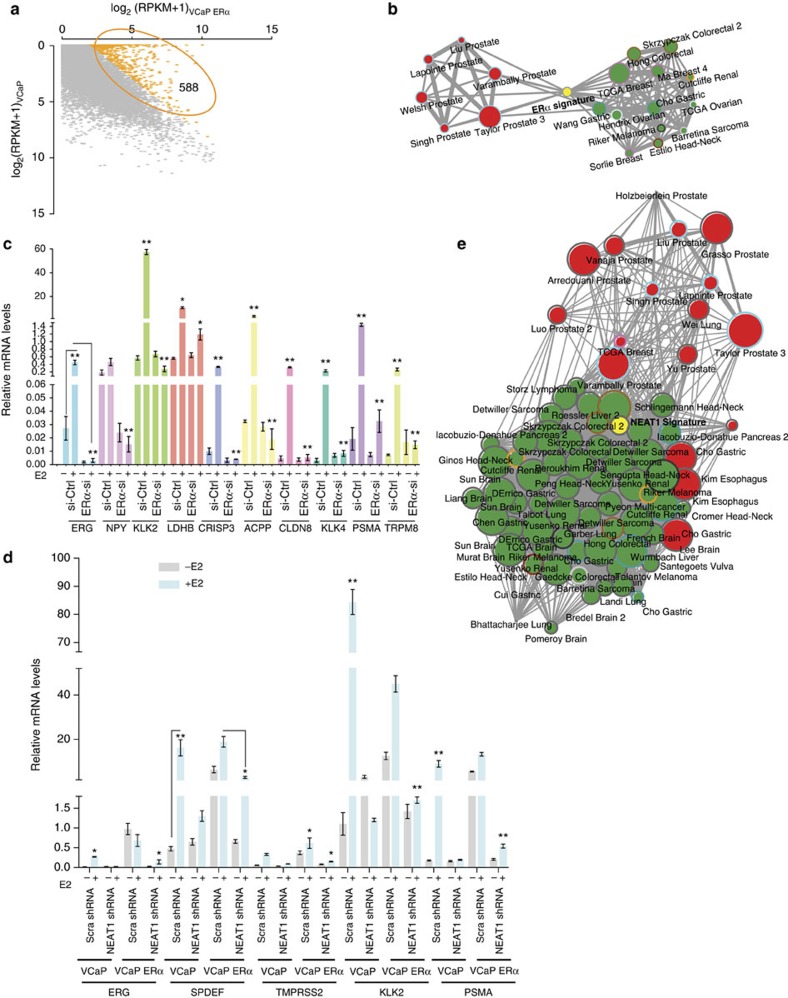
NEAT1 ERα signature correlates with prostate cancer. (**a**) Scatter plots for gene expression levels in VCaP ERα compared with VCaP cell lines. (**b**) Five hundred and eighty-eight genes that are overexpressed in VCaP ERα (log2-fold change >2) were used for Oncomine concept analysis across different cancer data sets (see Methods for detail). (**c**) qRT–PCR analysis of relative mRNA levels of ERα target genes in VCaP cells with knockout of ERα with and without E2 treatment. The target genes selected for validation are the ones that had the highest log2-fold difference in VCaP and VCaP ERα cell lines. Results are expressed as the mean±s.d. calculated from three independent experiments. Student’s *t*-test was performed (as indicated) for comparisons between −E2 and +E2 conditions for Ctrl siRNA and ERα-siRNA transfections, and *P<0.05 and **P<0.01 were considered statistically significant. A representative example is shown for ERG target expression. (**d**) qRT–PCR analysis of ERα target genes in VCaP cells with ERα overexpression and NEAT1 knockout with and without E2 treatment. Results are expressed as the mean±s.d. calculated from three independent experiments. Student’s *t*-test was performed for comparisons between −E2 and +E2 conditions for scrambled shRNA and NEAT1 shRNA transfections in VCaP and VCaP ERα cells, and *P<0.05 and **P<0.01 were considered statistically significant. A representative example is shown for SPDEF target expression. (**e**) Network representation of NEAT1 signature, derived from genes overexpressed in VCaP NEAT1 (NEAT1 signature) cells, across different cancer data sets using Oncomine concept analysis.

We next sought to understand the physiological role of NEAT1 and to determine the downstream targets of the ERα-NEAT1 axis in prostate cancer. We were particularly interested in identifying genes significantly deregulated in prostate cancer and positively correlated with ERα and NEAT1 expression. Transcriptome sequencing of VCaP and VCaP ERα cells and pairwise comparison revealed 588 genes to be upregulated in VCaP ERα cells (log2-fold change >2) (Supplementary Dataset 3 and Fig. 3a). We performed a comparative analysis of this 588 gene signature using Oncomine concept analysis. We focused on data sets from prostate cancer studies that included both prostate tumour and benign prostate tissues. The analysis revealed that the ERα gene signature was significantly upregulated in a number of prostate cancer data sets, but was downregulated in other non-prostate data sets, indicating that ERα regulates prostate cancer-specific genes (Fig. 3b and Supplementary Dataset 4).

To validate whether ERα targets identified by *in silico* analysis are dependent on cellular levels of ERα, we experimentally silenced ERα in VCaP cells using an small interfering RNA (siRNA) approach and determined transcript levels of ten target genes using qRT–PCR. The target genes selected for validation were those genes that demonstrated the highest log2-fold difference in VCaP and VCaP ERα cells. Results indicated that messenger RNA levels of the target genes selected were dependent on ERα (Fig. 3c), suggesting a distinct contribution of ERα in determining the transcriptional programme.

### NEAT1 is a downstream target in the ERα signalling pathway

After determining an ERα signature, we next investigated the potential role of NEAT1. Interestingly, knockout of NEAT1 compromised the expression of ERα target genes, suggesting that NEAT1 is not only a downstream target but also a mediator of ERα signalling in prostate cancer cells (Fig. 3d). To evaluate this further and to determine whether a functional synergy between ERα and NEAT1 pathways exists in prostate cancer cells, we performed RNA-seq of vector control and NEAT1-overexpressing VCaP cells to determine a NEAT1 signature. To achieve this, we limited our analysis to genes that were upregulated four-fold in NEAT1-expressing cells (Supplementary Dataset 5). Interestingly, the NEAT1 signature showed a strong correlation with the ERα signature genes (*q*=1.90E−120). Analysis of the top 1,000 genes of the NEAT1 signature revealed that this signature is upregulated in prostate cancer data sets when compared with other cancer data sets (Fig. 3e and Supplementary Dataset 4). Furthermore, the NEAT1 signature was also upregulated in all prostate cancer data sets (comparing benign versus PCa; odds ratio >2.0 and *P*<1 × 10^−6^) (Supplementary Fig. 3a).

We also queried Oncomine prostate data sets to identify genes whose mRNA levels correlate with those of NEAT1 (correlation coefficient >0.5). We compared this gene list with the ERα signature genes from our analysis in Fig. 3a and identified 155 genes in common. These 155 genes were also found to be upregulated in all prostate cancer data sets compared with other cancer data sets (only normal versus cancer data sets were considered; odds ratio >3.0 and *P*<1 × 10^−6^) (Supplementary Dataset 4 and Supplementary Fig. 3b).

**Figure 4:**
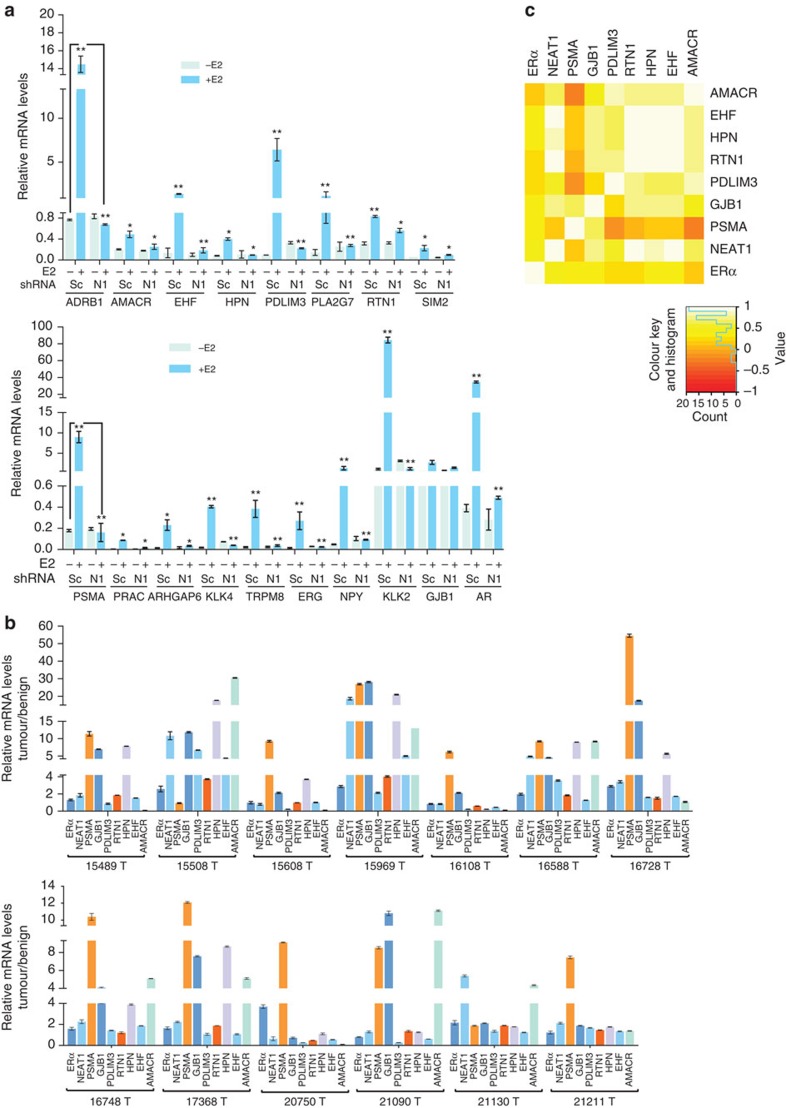
NEAT1 ERα signature is upregulated in prostate cancer. (**a**) Relative mRNA levels of genes nominated from analysis in Fig. 3b,e, analysed using qRT–PCR in parental VCaP cells transfected with scrambled (Sc) and NEAT1 shRNA (N1), respectively, with and without E2 (10 nM) treatment. Results are expressed as the mean±s.d. calculated from three independent experiments. Student’s *t*-test was performed for comparisons (relative mRNA levels of target gene expression) between −E2 and +E2 conditions for scrambled shRNA and NEAT1 shRNA transfections. A representative example is shown for ADRB1 and PSMA target expression. *P<0.05 and **P<0.01 were considered statistically significant. (**b**) Validation of expression of the top target NEAT1 ERα signature genes in a small matched patient cohort of 13 benign and 13 PCa, *n*=26. Results are expressed as the relative mRNA levels tumor/benign from two independent experiments. Error bars represent the range of data. (**c**) Heatmap shows the Spearman’s correlation results from **b**.

To determine whether the genes identified by *in silico* analysis are indeed influenced by NEAT1, we silenced NEAT1 in VCaP cells and determined transcript levels of potential target genes using qRT–PCR. We selected the top ten genes that were significantly correlated to NEAT1 expression across all prostate cancer concepts. As expected, mRNA levels of these selected target genes were indeed dependent on NEAT1, further confirming a definite role of NEAT1 in the transcriptional programme (Fig. 4a). In addition to cell lines, we also determined transcript levels of these ERα-NEAT1 signature-selected genes in a small patient cohort (*n*=26) of 13 matched benign and PCa, respectively. We observed that relative mRNA levels of these NEAT1-ERα signature-selected genes revealed significant upregulation in prostate cancer (Fig. 4b). We computed the log2-fold change of expression levels using the 13 paired tumour/benign samples for NEAT1 and for these selected genes. We then correlated the fold change values and observed a moderate-to-strong correlation between NEAT1 and the associated genes in clinical samples (Fig. 4c). Among these seven genes, prostate-specific membrane antigen (*PSMA*) and alpha-methylacyl-CoA racemase (*AMACR)* are well-known diagnostic and, in the case of PSMA, prognostic markers of prostate cancer progression^40^,^41^,^42^,^43^,^44^. Furthermore, knocking down ERβ did not alter expression of key signature genes in LnCaP, PC3, VCaP and NCI-H660 cells (Supplementary Fig. 3c), suggesting a non-redundant regulatory role for ERα.

### NEAT1 and chromatin regulation

**Figure 5:**
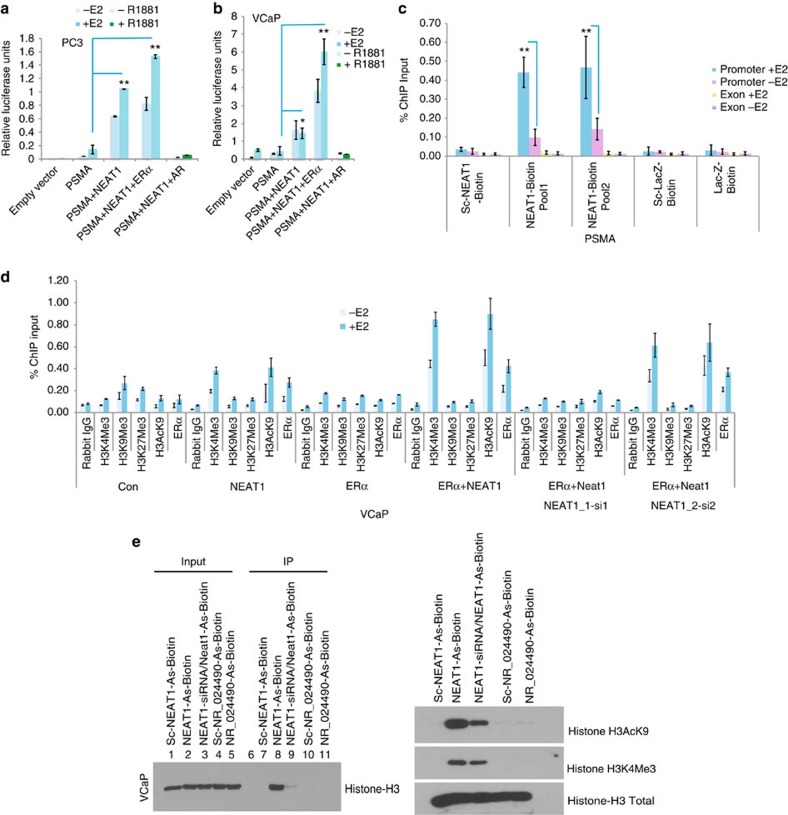
NEAT1 is a transcriptional regulator. (**a**,**b**) Promoter luciferase reporter assay shows that NEAT1 activates *PSMA* promoter in PC3 and VCaP cells. Cells were co-transfected with empty vector or PSMA luc and Renilla-luc reporter genes alone or with NEAT1, NEAT1+ERα and NEAT1+AR. Luciferase activity was measured 48 h post treatment with E2 (10 nM) or R1881 (1 nM). Results are expressed as the mean±s.d. calculated from three independent experiments. Student’s *t*-test was performed for comparisons (relative PSMA–luciferase activity) between −E2 and +E2 conditions for vector control, NEAT1 and NEAT1+ERα transfections in PC3 and VCaP cells. *P<0.05 and **P<0.01 were considered statistically significant. (**c**) Quantitative analysis of NEAT1 ChIRP in VCaP cells with or without E2 treatment (10 nM). Recruitment profiles of NEAT1 to PSMA are shown. Results are expressed as the percentage of input calculated from two independent experiments. Error bars represent the range of data. Results were reproducible between representative experiments. **P<0.01 was considered statistically significant. (**d**) Analysis of the chromatin landscape at the *PSMA* promoter performed by ChIP in VCaP cells alone or transected with NEAT1, ERα, NEAT1 ERα, NEAT1 ERα NEAT1_1 siRNA and NEAT1 ERα NEAT1_2 siRNA with and without E2 treatment. qPCR was performed with specific primers for the PSMA promoter. Results are expressed as the percentage of input calculated from two independent experiments. Error bars represent the range of data. Results were reproducible between representative experiments. (**e**) NEAT1 binds to Histone H3. 20 mer-biotinylated NEAT1 and NR_024490 antisense probes were used to immunoprecipitate NEAT1 and NR_024490 from nuclear lysates of VCaP cells using streptavidin magnetic beads. Immunoprecipitates from Streptavidin-IP were analysed on 15% gel and probed for Histone H3. NEAT1 is shown to also bind with active histone H3 modifications, including H3AcK9 and H3K4Me3.

To study the potential role of NEAT1 in regulation of target genes *in vivo*, we performed luciferase reporter assays using PSMA-luc as a candidate NEAT1 target. NEAT1 induced robust activation of the *PSMA* promoter in PC3 cells (Fig. 5a) and VCaP cells (Fig. 5b). These results prompted us to investigate whether NEAT1 is recruited to chromatin of target genes. We used the chromatin isolation by RNA purification (ChIRP) approach^45^ to pull down endogenous NEAT1 from VCaP cells. Analysis of the ChIRP data revealed that NEAT1 is recruited to the *PSMA* promoter, but not the downstream exon 1 (Fig. 5c). In addition to *PSMA*, we also tested NEAT1 recruitment to other target genes described in Figs 3c and 4a, and observed that in addition to *PSMA*, NEAT1 was also recruited to the promoter region of *GJB1* (Supplementary Fig. 4a). This suggests that NEAT1 transcriptionally regulates a compendium of genes known to be involved in prostate cancer progression. We hypothesized that NEAT1 might contribute to gene transcription by interacting with chromatin-modifying proteins and/or interacting with histones. Several recent studies support the view that lncRNAs recruit chromatin-modifying machinery^46^,^47^,^48^,^49^. To test this hypothesis, we analysed the chromatin landscape at the *PSMA* promoter and observed that NEAT1_1, and not NEAT1_2, facilitated gene transcription by promoting an active chromatin state (Fig. 5d). Overexpression of NEAT1_1 significantly increased active chromatin marks at the *PSMA* promoter (that is, H3K4Me3 and H3AcK9). Of note, ERα was not significantly recruited to the *PSMA* promoter when expressed alone. Overexpression of NEAT1_1 resulted in subsequent recruitment of NEAT1_1 and ERα to the *PSMA* promoter. These studies indicate that although NEAT1_1 may function as a chaperone for ERα and other chromatin-modifying machinery to target promoters, binding of ERα and/or recruitment to NEAT1_1 targets is not necessary for transcriptional activation.

As our data suggests that NEAT1 overexpression favours a chromatin landscape for active transcription, we investigated whether NEAT1 could directly interact with nucleosomal histones. Nuclear lysates from VCaP cells were used in an immunoprecipitation experiment with streptavidin beads coupled with either scrambled, antisense NEAT1, or antisense NR_024490 (another ERα lncRNA target) oligonucleotides. NEAT1 was found to specifically associate with histone H3 (Fig. 5e, left panel, lane 8) and the specificity of this binding is apparent when comparing lanes 7 and 9, which represent Streptavidin-IP using scrambled biotinylated oligos and Streptavidin-IP using antisense-NEAT1 oligos and nuclear lysates from NEAT1 siRNA-treated cells, respectively. As an additional negative control, we used scrambled and specific antisense oligos for a different lncRNA, NR_024490, another ERα target. The results indicate that NEAT1 can associate with chromatin via a specific interaction with histone H3. We also determined association of NEAT1 with active histone H3 modifications, including H3AcK9 and H3K4Me3 (Fig. 5e, right panel). Similar association patterns were seen for NEAT1 in NCI-H660 cells (Supplementary Fig. 4b).

To complement this finding, we performed RNA immunoprecipitation from VCaP ERα cells using anti-histone H3 and anti SNRNP70 (positive control) as the immunoprecipitating antibody. qRT–PCR showed robust binding of NEAT1 to histone H3 (Supplementary Fig. 4c). The positive control U1 small nuclear RNA showed high enrichment in the immunoprecipitate with SNRNP70. To further confirm the specificity of NEAT1 binding to histone H3, we performed a streptavidin–biotin pull-down assay in VCaP and VCaP ERα cells with and without E2 (Supplementary Fig. 4d). These data suggest that NEAT1 can directly interact with the histone H3 component of chromatin.

### NEAT1 promotes prostate tumorigenesis

**Figure 6:**
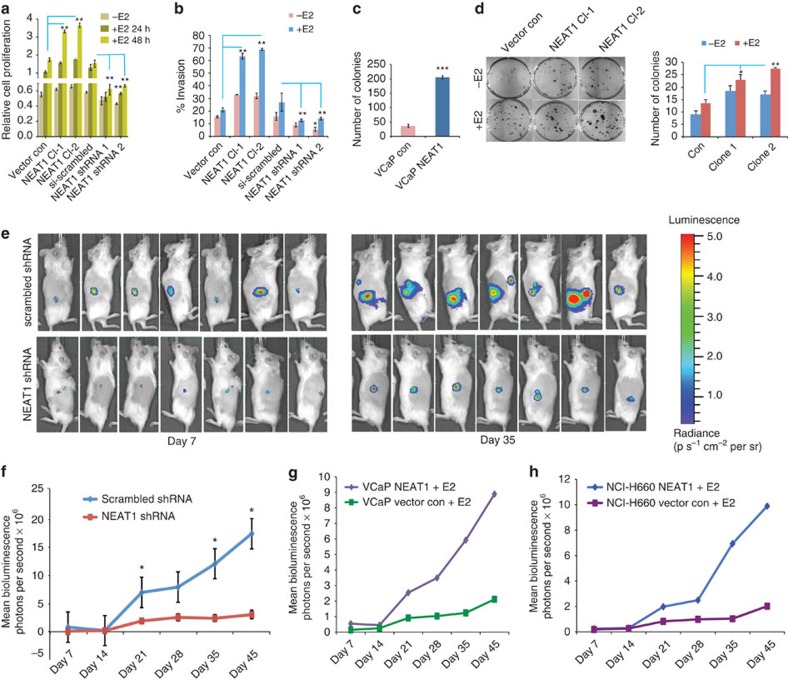
NEAT1 is a driver of oncogenic cascade. (**a**) Cell proliferation assays were performed in VCaP vector control, NEAT1-overexpressing cells and also in si scrambled and NEAT1 knockout cells with or without E2 treatment (10 nM) at 24 and 48 h time points. Results are expressed as the mean±s.d. calculated from three independent experiments. Student’s *t*-test was performed for comparisons (relative cell proliferation) between E2 conditions for vector control, NEAT1 Cl-1, and NEAT1 Cl-2 and E2 conditions for si-scrambled, Neat1-shRNA1 and shRNA2 transfections. **P<0.01 was considered statistically significant. (**b**) Quantitative bar chart for depicting percentage of cells invaded at the completion of invasion assays performed in VCaP vector control, NEAT1-overexpressing cells and also in si scrambled and NEAT1 knockout cells with or without E2 treatment (10 nM). Results are expressed as the mean±s.d. of three independent experiments. *P<0.05 and **P<0.01, Student’s *t*-test. (**c**) Soft agar assays were performed with VCaP control and NEAT1-expressing cells. Quantitative bar-plot analysis of stained colonies at 21 days are shown. Results are expressed as the mean±s.d. of three independent experiments. ***P<0.001, Student’s *t*-test. (**d**) Colony-forming assay were performed in VCaP vector control, NEAT1-overexpressing cells with or without E2 treatment (10 nM). The right panel depicts the number of colonies at 21 days. Results are expressed as the mean±s.d. calculated from three independent experiments. *P<0.05 and **P<0.01, Student’s *t*-test. (**e**) VCaP ERα cells expressing con shRNA luciferase (luc) and NEAT1 shRNA luc were injected subcutaneously into the flank of male NOD-SCID mouse. Bioluminescent imaging on Day 7 and Day 35 in the VCaP ERα scrambled shRNA (top panel) and VCaP ERα NEAT1 shRNA (bottom panel) injected mice is shown. (**f**) Growth curve for the tumours monitored upto 45 days. Results are expressed as the mean±s.d. calculated from three independent experiments. *P<0.05, Student’s *t*-test. (**g**,**h**) VCaP and NCI-H660 vector control and NEAT1-overexpressing cells were injected subcutaneously into the flank of male NOD-SCID mouse. Bioluminescence imaging monitored the tumour growth. Growth curve for the tumours monitored upto 45 days is shown; VCaP (**g**) and NCI-H660 (**h**).

To better understand the physiological role of NEAT1 in the context of ERα in prostate cancer, we first determined the levels of NEAT1 in VCaP cells overexpressing ERα (Supplementary Fig. 5a). Further, we generated stable VCaP and VCaP ERα cell lines that overexpress NEAT1 (Supplementary Fig. 5b). We also knocked down NEAT1 in VCaP and VCaP ERα-expressing cells by stably expressing NEAT1 shRNA targeting two different regions of NEAT1 and non-targeting shRNA (Supplementary Fig. 5c). Although overexpression of NEAT1 significantly increased proliferation and cell invasion, knockdown of NEAT1 significantly decreased proliferation and the invasive properties of the cells (Fig. 6a,b).

Soft agar assays were performed in both VCaP and VCaP NEAT1 cells. Colonies were monitored over a period of 21 days. Overexpression of NEAT1 resulted in a significantly higher number of viable colonies (Fig. 6c). Colony-forming assays performed in NEAT1 clones in VCaP cells with and without E2 demonstrated that E2 treatment in NEAT1-overexpressing cells significantly increased the number of colonies (Fig. 6d). These *in vitro* assays establish an oncogenic role for NEAT1.

To further validate the oncogenic role of NEAT1, we extended our studies to an *in vivo* model system. We performed xenograft studies in NOD-SCID mice. The mice were treated with time-release oestrogen pellets. They were divided into two groups and one group was implanted subcutaneously with VCaP ERα cells expressing control shRNA luciferase reporter, and the other group with VCaP ERα cells expressing NEAT1 shRNA luciferase reporter. The mice from both groups were imaged weekly for luciferase activity and Fig. 6e shows the bioluminescent signals at day 7 and day 35. The tumour growth was monitored weekly for 45 days and was found to be significantly lower in the NEAT1 shRNA-expressing group compared with the control group (Fig. 6f). The tumours were excised and weighed, and the NEAT1 shRNA group had significantly smaller tumours (Supplementary Fig. 6a,b). We confirmed the efficacy of the shRNA *in vivo* by measuring the NEAT1 and ERα levels in the tumours (Supplementary Fig. 6c).

To further substantiate our hypothesis that NEAT1 plays a role in tumorigenesis, we repeated the experiment in athymic nude mice using VCaP control and VCaP NEAT1-overexpressing cells, as well as NCI-H660 and NCI-H660 NEAT1-overexpressing cells. In both these experiments, a significantly higher tumour growth was seen in the NEAT1-overexpressing cells (Fig. 6g,h and Supplementary Fig. 6d,e) further confirming its oncogenic potential. qRT–PCR analysis confirmed an increased expression of the NEAT1 signature genes in VCaP NEAT1 xenografts compared with control VCaP xenograft tissue (Supplementary Fig. 6f).

### NEAT1 is associated with therapeutic resistance

**Figure 7:**
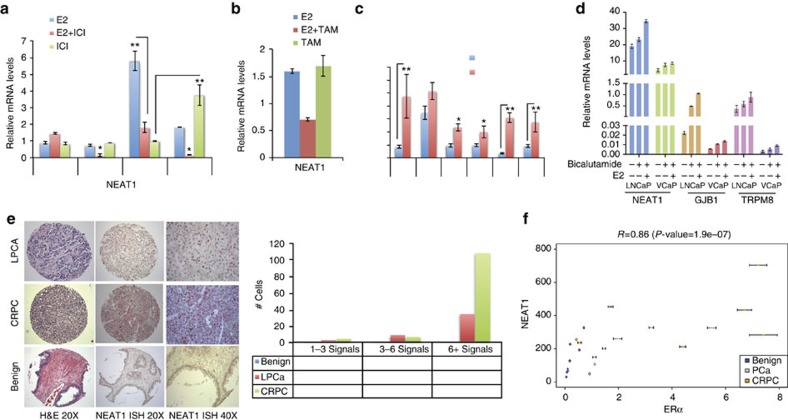
NEAT1 in therapy resistance. (**a**) NEAT1 expression in VCaP cells treated with E2 (10 nM) at different time points alone, E2+ICI (10 nM+10 μM) or ICI (10 μM) alone. Results are expressed as the mean±s.d. of three independent experiments. *P<0.05, **P<0.01, Student’s *t*-test. (**b**) NEAT1 expression in VCaP cells treated with E2, E2+4OHT (10 nM+10 nM) and 4OHT (10 nM) alone for 48 h. (**c**) NEAT1 expression in VCaP cells treated with or without E2 (10 nM) or E2+Enzalutamide (10 nM+10 μM) at different time points. Results are expressed as the mean±s.d. of three independent experiments. *P<0.05 and **P<0.01, Student’s *t*-test. (**d**) qRT–PCR analysis of NEAT1, GJB1 and TRPM8 in LnCaP and VCaP control cells, with bicalutamide treatment (10 μM) alone or in combination with E2 (10 nM) for 48 h. Results are expressed as the means±s.d. of three independent experiments. Results were reproducible between representative experiments. (**e**) Representative image for RNA ISH of NEAT1 in benign, localized PCa and in advanced disease (top panel). Quantification for the RNA ISH signals shown in the bottom using RNA Spot Studio. (**f**) Scatter plot showing the correlation between ERα and NEAT1 expression by qRT-PCR in nine cases of benign prostate, seven PCa and seven CRPC. Pearson’s correlation coefficient *R*=0.86 (*P*-value=1.9e−07).

The study presented so far shows that ERα establishes an oncogenic cascade and that NEAT1 functions as a downstream mediator of ERα signalling. The ERα-NEAT1 axis is functional both in AR-positive and -negative cell lines, and drives prostate carcinogenesis. We hypothesized that targeting NEAT1 using mechanisms that can constrain ERα might represent a novel therapeutic strategy in prostate tumours that are resistant to anti-androgen therapy. To test this hypothesis *in vitro*, we evaluated the effect of anti-oestrogens and anti-androgens on NEAT1 levels in prostate cancer cell lines. As shown in Fig. 7a,b, NEAT1 expression is constrained when cells are treated with the ERα antagonists ICI 182,780 (ICI) and 4-hydroxy tamoxifen (4OHT) in combination with E2. Intriguingly, treatment of ICI and 4OHT alone for longer periods can enhance NEAT1 expression (Fig. 7a,b). We observed similar results with AR antagonists enzalutamide and bicalutamide (Fig. 7c,d). These results provide compelling evidence to evaluate NEAT1 levels in advanced CRPC cases. RNA-fluorescent ISH analysis of benign and advanced prostate tumours, including CRPC and NEPC tumour tissue samples, illustrated significantly upregulated NEAT1 levels in advanced prostate cancer, with enhanced focal staining throughout the tumour tissue (Fig. 7e). We also screened nine cases of benign prostate, seven PCa and seven CRPC (Supplementary Table 2) for NEAT1 and ERα expression by qRT-PCR (Fig. 7f), and both NEAT1 and ERα levels were significantly higher in the CRPCs. We determined the correlation between NEAT1 and ERα expression by estimating the Pearson’s correlation coefficient *R*. The results indicate a strong positive correlation: *R*=0.86 (*P*-value=1.9e−07). Taken together, our results present a novel role for the non-coding transcriptome in cancer-favourable adaptations.

### NEAT1 is associated with aggressive prostate cancer

Given the importance of NEAT1 in promoting tumorigenesis both *in vitro* and *in vivo*, we sought to determine the relationship between NEAT1 levels and prostate cancer clinical outcomes in 594 patients from two radical prostatectomy cohorts with long-term clinical follow-up from the Mayo Clinic^35^,^36^. Supplementary Table 1 contains the patient characteristics of men who underwent radical prostatectomy at the Mayo Clinic Comprehensive Cancer Center between 1987 and 2001 for clinically localized prostate cancer.

**Figure 8:**
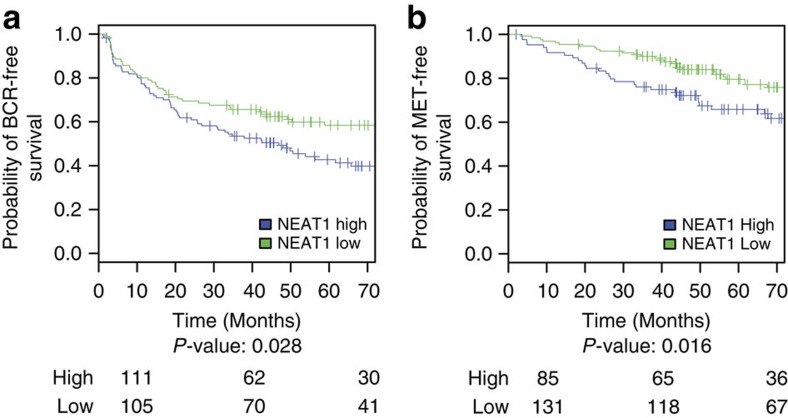
NEAT1 overexpression is associated with aggressive prostate cancer. (**a**,**b**) KM curves showing (**a**) BCR-free survival and (**b**) MET-free survival for NEAT1 low and high expression groups of samples from the Mayo case–cohort data set^35^ (*n*=216). The cut points to define high and low NEAT1 expression were selected using patients from the Mayo nested case–control data set (*n*=378)^58^ by maximizing the product of the sensitivity and specificity for each endpoint. The number of patients at risk for each group is shown beneath the plot.

We assessed the prognostic potential of NEAT1 expression using several statistical measures and correlating it with biochemical recurrence (BCR) and metastasis (MET), prostate cancer-specific mortality (PCSM) and GS>7. To evaluate endpoints of disease aggressiveness and progression based on NEAT1 expression, Kaplan–Meier (KM) analysis was performed for the BCR and MET endpoints. The resulting KM curves (Fig. 8a,b) demonstrate that patients with higher NEAT1 expression have significantly worse outcomes for both BCR (*P*-value: 0.028) and MET events (*P*-value: 0.016).

Patient risk discrimination based on the expression profile of NEAT1 was assessed by area under the receiver operating characteristic curve with 95% confidence intervals (Supplementary Fig. 7). NEAT1 significantly segregates patients who exhibited BCR, MET, PCSM and GS>7.

**Figure 9:**
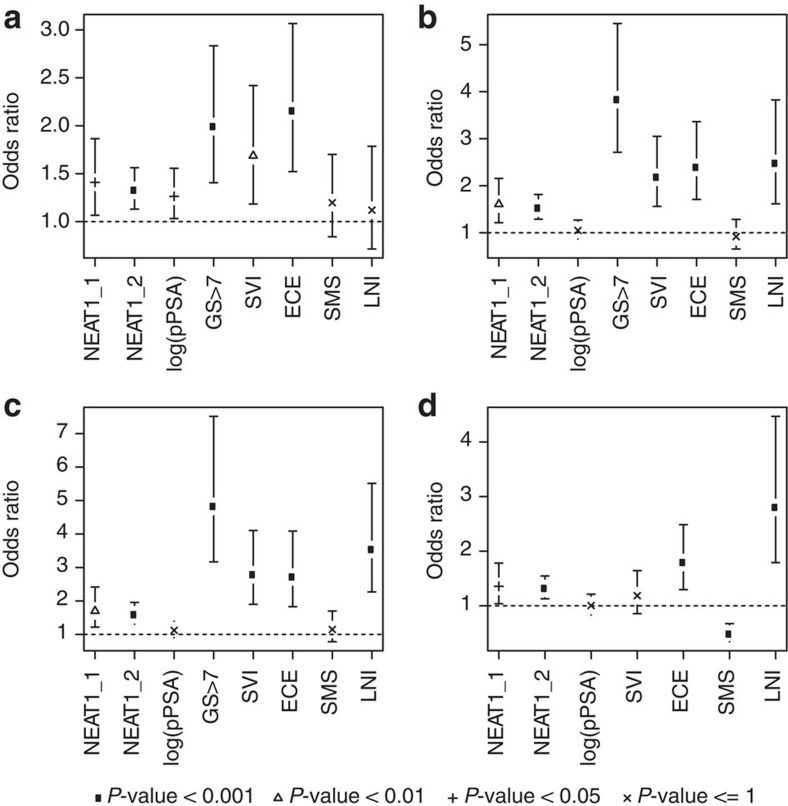
NEAT1 is a strong prognosticator of prostate cancer. (**a**–**d**) Univariable forest plots comparing the expression of NEAT1’s short (NEAT1_1) and long isoform (NEAT1_2) to clinicopathologic variables in the pooled Mayo cohort (*n*=594) (**a**) BCR, (**b**) MET, (**c**) PCSM and (**d**) GS>7. Pathological tumour stage 3 or greater (pT3+), lymph node invasion (LNI), surgical margin status (SMS) positive, seminal vesicle invasion (SVI), extra capsular extension (ECE), preoperative PSA (pPSA), adjuvant hormone therapy and adjuvant radiation therapy are shown.

To further compare NEAT1’s prognostic ability to other clinicopathologic variables, univariable odds ratios were computed for the BCR, MET, PCSM and GS>7 endpoints (Fig. 9). NEAT1 was significantly prognostic for segregating high-risk from low-risk patients for each of the endpoints (*P*<0.05). Further multivariate analysis adjusting for adjuvant radiation and hormone treatment, in addition to the other clinicopathological variables assessed, also demonstrates that NEAT1 was significantly prognostic for BCR, MET and GS>7, supporting NEAT1 as a prognostic biomarker for aggressive prostate cancer independent of common clinical and pathologic variables (Supplementary Fig. 8). Overall, these results show that NEAT1 is significantly prognostic for several clinically relevant endpoints.

## Discussion

The tissue-specific role of ERα in breast and other gynaecological malignancies is well understood. Interestingly, ERα is expressed in all prostate cancers, including those that lack AR expression, while it is absent in normal prostate epithelium. Studies from our and other laboratories have examined the relevance of ERα in prostate cancer^4^,^50^,^51^,^52^. ERα-mediated regulation of oncogenic TMPRSS2-ERG fusion and oestrogen regulation of the *EBAG9* gene, which confirms aggressive behaviour of prostate cancer, are noted examples that suggest a functional ERα-signalling pathway exists in prostate cancer. From a clinical perspective, the association of a polymorphism in ERα with prostate cancer with a favourable GS or cancers of late onset has also been reported^53^. These initial observations prompted us to evaluate whether re-expression of ERα and the establishment of an alternate nuclear receptor-signalling axis (that is, ERα versus AR) in prostate cancer cells could represent an adaptive mechanism to evade AR-directed therapies.

Analysis of global ERα recruitment in prostate cancer cells using a ChIP-seq approach revealed that ERα is preferentially recruited to intergenic regions of the prostate genome. Comparison of binding profiles with transcriptome sequencing data suggested that ERα drives expression of non-coding transcripts. These results led us to analyse the functional consequences of ERα recruitment to non-coding regions. From a large compendium of ERα-regulated non-coding transcripts, we selected NEAT1 for a detailed biochemical and *in vivo* evaluation, based on an *in silico* approach that demonstrated a strong association of NEAT1 with prostate cancer progression. We show that ERα transcriptionally regulates NEAT1. NEAT1 is recruited to the promoter of several key target genes and induces an active chromatin state favourable for transcription. Our studies indicate that ERα does not function as a molecular chaperone to guide NEAT1 to target chromatin; rather, we suspect that a complex proteome of chromatin-interacting proteins interacts with and guides NEAT1 to promoter targets. Interestingly, both ERα and NEAT1 signalling were refractory to AR inhibitors and the lack of AR or ERβ, thus indicating a functional specialization of the ERα-NEAT1 axis for prostate cancer progression. Furthermore, introduction of cells overexpressing NEAT1 could clearly induce prostate cancer progression in experimental animal models.

The current study opens up a new arena of alternative mechanisms of tumorigenesis by ERα in prostate cancer. We show that ERα regulates NEAT1 lncRNA with distinct chromatin regulatory functions. Large-scale bioinformatic analysis of SAGE libraries has identified NEAT1 as one of the differentially regulated lncRNAs between some types of cancer and normal tissue^54^. However, its possible role in promoting tumorigenesis has never been explored. We show here that NEAT1 regulates expression of prostate cancer genes by altering the epigenetic landscape at target gene promoters to favour transcription. A closer examination of NEAT1 revealed a previously uncharacterized role in recognition of modified histones. We have not tested whether NEAT1 is a reader of multiple histone H3 post-translational modifications (acetylation, methylation and so on) and our laboratory is actively pursuing this intriguing question. NEAT1 expression independently was sufficient to activate prostate cancer genes in an AR-independent manner. Further, our results confirmed an oncogenic role for NEAT1 in an experimental animal model of prostate cancer and in cell culture models.

Molecular sieving of the net non-coding transcriptome using comprehensive bioinformatic approaches and wet-lab validation over a decade has indicated that the non-coding transcriptome has a regulatory role beyond the speculated ‘transcriptional noise’ and a direct influence on the coding transcriptome and biologic homeostasis. We observed that several lncRNAs such as NEAT1 respond to cellular cues and ligand signalling in a manner reminiscent of the coding transcriptome. Thus far, the literature on NEAT1 has focused on its architectural role in forming subnuclear paraspeckles^27^. Our results indicate a role for NEAT1 beyond that of paraspeckles. It would be interesting in the future to reconcile how the formation of paraspeckles in the inter-chromosomal space ties in with the role of NEAT1 in activating gene expression at promoters. Our lab continues to pursue some of these unanswered questions to better understand the role of NEAT1.

**Figure 10:**
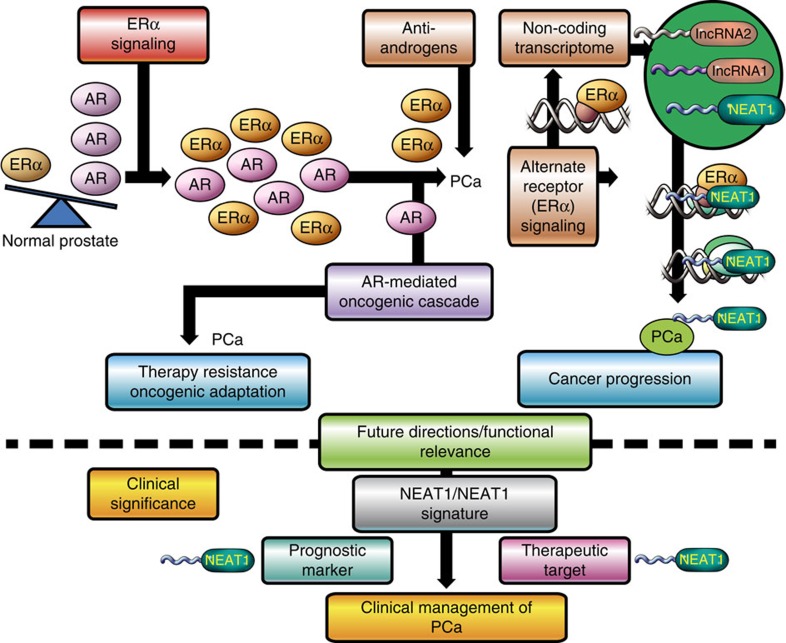
Model for NEAT1 function in prostate cancer. Functional ERα signalling in prostate cancer modulates expression of the lncRNA NEAT1. Prostate epithelial cells positive for NEAT1 have an oncogenic advantage and are refractile to androgen inhibitors or androgen ablation therapy. NEAT1, a histone interacting lncRNA and transcriptional regulator, is recruited to promoters of several prostate cancer-specific genes. NEAT1 can modulate the epigenetic landscape of target promoters and maintains expression of AR-dependent and -independent genes. The selection of alternate nuclear receptor signalling is a novel hallmark of prostate cancer progression.

Our identification of an ERα-NEAT1 axis illustrates a mechanism whereby prostate cancer cells may develop therapeutic resistance through positive selection of an alternate nuclear receptor signalling pathway in the absence of AR or during androgen ablation therapy (Fig. 10). However, we cannot exclude the presence of other NEAT1-interacting chromatin factors. This is the subject of ongoing investigation.

From a clinical perspective, our studies indicate for the first time that NEAT1 is significantly prognostic for several clinically relevant endpoints. In prostatectomy specimens from two large cohorts, high NEAT1 expression was associated with a significant increase in both biochemical and metastatic recurrence rates compared to those with low NEAT1 expression.

In summary, this study provides important insights into a unique mechanism of ERα regulation in prostate cancer and identifies NEAT1 as a novel prognostic marker and potential therapeutic target in this disease. Although our studies have identified a previously unexplored function of ERα in regulating lncRNAs, it is also the first of its kind to demonstrate transcriptional regulation of lncRNAs by an alternative steroid receptor in prostate cancer. We propose that NEAT1 is directly involved in modulation of the phenotype of a leading disease. Combinatorial targeting of NEAT1 and AR may represent a unique therapeutic regimen within a subset of patients with advanced prostate cancer.

## Methods

### Cell culture and treatments

LnCaP and PC3 cells were grown in RPMI 1640 (Invitrogen) and supplemented with 10% fetal bovine serum (FBS) and 1% penicillin–streptomycin. RWPE1 cells were grown in Keratinocyte Serum Free Medium Kit (Gibco, 17005-042). VCaP and DU145 cells were grown in DMEM (Invitrogen) and supplemented with 10% FBS with 1% penicillin–streptomycin. NCI-H660 cells were grown in RPMI 1640 supplemented with 0.005 mg ml^−1^ insulin, 0.01 mg ml^−1^ transferrin, 30 nM sodium selenite, 10 nM hydrocortisone, 10 nM β-estradiol, 5% FBS, 1% penicillin–streptomycin and an extra 2 mM of L-glutamine (for a final concentration of 4 mM). For cell treatments in several experiments, we used 10–100 nM β-estradiol (Sigma Aldrich, St Louis, MO), 10 μM Enzalutamide (Astellas), 10 μM bicalutamide (Sigma Aldrich), 1–10 nM R1881 (PE Biosystems), 10–100 nM 4OHT (Sigma Aldrich) and 1–10 μM ICI (Tocris Bioscience).

### Plasmids, siRNAs and transfection

Plasmids, pcDNA 3.1, pcDNA3.1-ERα, pcDNA 3.1 AR, piLenti-GFP, piLenti-NEAT1 siRNA-GFP (set of four, sequences provided in Supplementary Table 3), iLenti-si-scrambled, pLenti-bicistronic-luc-NEAT1 were used. siRNAs for ERα, ERβ, AR, NEAT1 and NEAT1_2 were used, and the sequence is provided in Supplementary Table 3. For the mammalian expression vectors, Lipofectamine 3000 (Invitrogen) and Lonza nucleofection were used for transfection. The stable clones for NEAT1 overexpression as well as the scrambled and NEAT1 shRNA-expressing cells were generated by using the lentiviral vectors and by selection in puromycin.

### Identification of ERα-regulated lncRNA

A set of known lncRNAs was generated from various data sources: RefSeq: GENCODE v7, - ncRNA.org and lncRNAdb^55^ (see Supplementary Material) and those that were at least 200 nt long were selected, resulting in 12,483 lncRNAs. These lncRNAs were characterized according to their potential of being regulated by ERα by using ERα-binding sites information from ChIP-seq experimental data. Moreover, several histone marks were considered to provide evidence of transcription, including H3K4me3 and H3K36me3 (details in Supplementary Material).

### Differential expression analysis

To prioritize the experimental validation of lncRNAs, a pair-wise differential expression analysis was performed on the expression values determined by paired-end transcriptome sequencing of 73 samples (26 benign prostate, 40 PCa and 7 NEPC). A pair-wise Wilcoxon test was performed and all *P*-values were corrected for multiple hypotheses testing using Benjamini–Hochberg^56^ (details in Supplementary Material).

### ERα and NEAT1 signature via Oncomine concept analysis

RNA sequencing was done for VCaP and VCaP ERα-expressing cells, as well as in vector control and NEAT1-overexpressing VCaP cells (detailed in Supplementary Methods). The expression of the genes was computed and those genes with a log2-fold change >2 were selected. Results are reported in Supplementary Datasets 3 and 5. Five hundred and eighty-eight genes were found to be overexpressed in VCaP ERα cells. A custom concept of this gene list was generated in Oncomine (Supplementary Dataset 4). Similarly, genes from the VCaP NEAT1 group with a log2-fold change >2 were selected and a custom concept was built in Oncomine using the top 1,000 genes from NEAT1 signature (Supplementary Dataset 4). The significantly associated tumour versus normal concepts with odds ratio >2.0 and *P*<1 × 10^−6^ considering tumour versus normal analysis was determined. The resulting concepts and associations are represented through a concept network using Cytoscape version 2.8.2. Each node represents a concept to which the signature is associated at a >3-fold odds ratio for ERα signature and >2-fold odds ratio for NEAT1 signature. Node size reflects the concept size, that is, the number of genes in each concept; red and green colours represent correlation with over- or underexpressed genes in the concept, respectively; and edge thickness represents the odds ratio of the association between concepts, ranging from 1.4 to 29.9 and 1.2 to 637 for ERα and NEAT1 signatures, respectively. The border colour of each node represents the tumour type. The layout of the network is based on the Edge-weighted spring-embedded algorithm.

### Luciferase reporter assays

For ERE luciferase assays, VCaP cells were transiently transfected with the (ERE)3-SV40-luc reporter plasmid and/or ERα and/or AR, as well as an internal control construct pRL harbouring the renilla luciferase gene. VCaP cells were also transfected with empty vector or *NEAT1* promoter (1+2) luciferase reporter constructs alone or with ERα, as well as an internal control construct pRL harbouring renilla luciferase gene. To determine the PSMA reporter activity, 293T and PC3 cells were co-transfected with empty vector or PSMA luc and Renilla-luc reporter genes alone or with NEAT1, NEAT1+ERα or NEAT1+AR.

Twenty-four hours post transfection, the media was changed to 5% charcoal-stripped media and the cells indicated were treated with E2 (10 nM) or R1881 (1 nM) for 14 h. At 48 h, cells were lysed with passive lysis buffer and luciferase activities were measured using the dual luciferase system (E1910, Promega) and normalized with renilla luciferase activity.

### RNA ISH for NEAT1

RNA ISH for NEAT1 was performed on five benign, five PCa and three CRPC cases using kits and probes designed by Advanced Cell Diagnostics. Briefly, the single-colour chromogenic detection assay uses pairs of specially designed oligonucleotide probes that through sequence-specific hybridization, recognize both the specific target NEAT1 RNA sequence and the signal amplification system. Unique target probe oligonucleotides were designed to hybridize in tandem to the target RNA. Cross-hybridization to other sequences is minimized by screening against the entire human RNA sequence database.

The signal amplification system consists of the pre-amplifier, amplifier and enzyme-conjugated label probe, which assemble into a tree-like complex through sequential hybridization. Signal amplification occurs at target sites bound by probe pairs only. Nonspecific off-target binding by single probes does not result in signal amplification.

All steps of NEAT1 RNA ISH staining of the slides are performed manually, optimized in tissue microarrays. Briefly, formalin-fixed, paraffin-embedded unstained tissue sections (5 μm) were mounted on positively charged microscopic glass slides, deparaffinized in xylene and rehydrated through a series of alcohols. The rehydrated sections were treated with 3% hydrogen peroxide at room temperature (RT) for 10 min to block endogenous peroxidase. Sections were then boiled in 1 × citric buffer (10 nmol l^−1^ Nacitrate, pH 6.0) for 15 min and incubated with protease (2.5 mg ml^−1^; Sigma Aldrich) at 40 °C for 30 min. The slides were hybridized sequentially with target probes (20 nmol l^−1^) in hybridization buffer A (6 × saline sodium citrate (SSC) buffer (1 × SSC is 0.15 mol l^−1^ NaCl and 0.015 mol l^−1^ Na-citrate), 25% formamide, 0.2% lithium dodecyl sulfate (LDS) and blocking reagents) at 40 °C for 2 h, signal pre-amplifier in hybridization buffer B (20% formamide, 5 × SSC, 0.3% LDS, 10% dextran sulfate and blocking reagents) at 40 °C for 30 min, amplifier in hybridization buffer B at 40 °C for 30 min and horseradish peroxidase- or alkaline phosphatase-labelled probes in hybridization buffer C (5 × SSC, 0.3% LDS and blocking reagents) at 40 °C for 15 min.

Hybridization signals were detected under bright-field microscope as red colorimetric staining (using Fast Red chromogen, BioCare Biomedical, Concord, CA) followed by counterstaining with haematoxylin. Signals were granular and discrete red signals corresponding to individual lncRNA targets. The signals were scored using the RNA Spot Studio software.

### Chromatin immunoprecipitation

All ChIP experiments were carried out using Millipore EZ-Magna ChIP kit (catalogue number 17–10086). Briefly, 5–10 × 10^6^ cells were cross-linked with 1% formaldehyde for 10 min at rRT. The cross-linking was then quenched with 0.125 M glycine. Chromatin was sonicated in the lysis buffer to 300–500 bp and the extraction of ChIP DNA was done as per the kit protocol. Antibodies used include ERα (AC-066-100, diagenode, 5 μg), AR (06–680, Millipore, 5 μg), H3K4me3 (ab8580, Abcam, 5 μg), trimethylated lysine 9 of histone H3 (ab8898, Abcam, 5 μg), H3K36me3 (ab9050, Abcam, 5 μg), trimethylated lysine 27 of histone H3 (07–449, Millipore, 5 μg) and Ace-H3 (no. 06–599, Millipore, 5 μg).

ERα ChIP was also performed in cross-linked VCaP cells with E2 treatment for 0, 14 and 48 h. In VCaP ERα cells, E2 treatment was for 6, 14 and 48 h. The primer sequences are provided in Supplementary Table 4.

### Chromatin isolation by RNA purification

ChIP for NEAT1 was done in VCaP control and NEAT1-expressing cells, with and without E2 treatment using the ChiRP protocol^45^. Briefly, biotin TEG antisense oligos were generated using singlemoleculefish.com for NEAT1, Lac Z and scrambled NT NEAT1. The NEAT1 probes were divided into two pools. Cells cross-linked in 1% glutaraldehyde were lysed and sonicated. The biotinylated probes were hybridized followed by RNA and DNA isolation. qPCR was performed on the DNA samples. Probe sequences are described in Supplementary Table 5.

### RNA ISH for NEAT1 on cell lines

Cells were grown on a 15 mm, poly-L-lysine-coated glass coverslip. At ~70% confluence, cells were serum starved in 8% charcoal-stripped media for 48 h, followed by 48 h treatment with 10 nM E2. At the end of treatment, cells were fixed in 4% formaldehyde, dehydrated by an ethanol gradient (50–100%) and stored at −20 °C. For the hybridization assay, cells were rehydrated by an ethanol gradient (100–50%) into PBS. Between subsequent steps, cells were washed with PBS. The Affymetrix QuantiGene ViewRNA ISH cell assay kit was used for NEAT1 staining. Cells were permeabilized by 5 min incubation at RT in Detergent Solution QC and digested for 10 min at RT by Protease QS (1:4,000 in PBS). Next, the target-specific Probe Set (1:100 in Diluent QF) was allowed to hybridize for 3 h at 40±1 °C. Between subsequent steps, cells were washed by soaking in Wash Buffer. Sequential hybridization steps were conducted for signal amplification—PreAmplifier Mix (1:25 in Diluent QF), Amplifier Mix (1:25 in Diluent QF) and Label Probe Mix (1:25 in Diluent QF), each incubated 30 min at 40±1 °C. After two 10-min washes in Wash Buffer, nuclei were stained with 4',6-diamidino-2-phenylindole and cover slips were mounted to slides with Prolong Gold Antifade Reagent (Life Technologies) for visualization.

### Proliferation assay

Cell proliferation was assessed using the CyQUANT NF cell proliferation assay kit (Life Technology). Cells were seeded in 96-well plates at 3–4 × 10^4^ cells per well. Cells were incubated in DMEM media with 10% FBS for 24 h. The cells were then serum starved in 8% charcoal-stripped DMEM medium for 48 h followed by E2 treatment at 10 nM concentration for indicated time points. The media was then aspirated and replaced with the dye-binding solution followed by incubation for 30–60 min. The fluorescence was then measured in a microplate reader using excitation at 485±10 nm and fluorescence detection at 530±15 nm. The assay was performed in triplicates.

### Invasion assay

The CHEMICON cell invasion assay kit (EMD MIlipore) was used for determining the cell invasion. Cells were serum-starved for 48 h and then seeded at a density of 2 × 10^5^ cells per well in the upper well of the invasion chamber. Five hundred microlitres of phenol red-free DMEM media supplemented with 8% charcoal-stripped serum and 10 nM E2 was added to the lower chamber. After 48-h incubation, the invaded cells were stained by dipping the inserts in the staining solution for 20 min. The stained cells were then dissolved in 10% acetic acid and transferred to a 96-well plate for colorimetric reading of optical density at 560 nm.

### Migration assay

The Cell Biolabs Inc. Radius 96-Well Cell Migration Assay was used to determine cell migration. Cells were serum starved for 48 h, then seeded to a pretreated (incubated 20 min in Radius Gel Pretreatment solution and washed with Radius Wash Solution) Radius 96-Well Plate at a density of 8 × 10^4^ cells per well with or without E2 (10 nM). After 24 h incubation, the Radius Gel Spot was removed via the Radius Gel Removal Solution and pre-migration images were captured. After 24-h incubation, cells were stained with Cell Stain Solution and post-migration images were captured for analysis using the CellProfiler Cell Image Analysis Software (Broad Institute).

### Statistical analysis

The Wilcoxon test was employed with Benjamini–Hochberg^56^ correction for multiple hypotheses for pair-wise comparisons for differential expression analysis. The *χ*^2^-test was used for comparison of proportions and the Pearson’s correlation was used to compare the expression of selected genes. For qRT–PCR, we computed the Delta CT value according to the ABI qPCR guidelines as described in Supplementary Methods. To compare qPCR data, a Student’s *t*-test was employed. Median rank statistics results are reported for analyses with the Oncomine data sets^57^.

### Analysis of Mayo clinic cohort

Affymetrix HuEx microarrays were used to analyse NEAT1 expression in two post-radical prostatectomy cohorts from the Mayo Clinic. Details on tissue preparation, RNA extraction, amplification, hybridization and clinical characteristics for these cohorts have been described previously^35^,^36^. Both cohorts were filtered using the same criteria (patient either exhibiting pre-operative prostate-specific antigen >20 ng ml^−1^, GS ≥8, pT3b or GPSM^58^ score ≥10) to increase the homogeneity of patient characteristics. The two sets were pooled to improve analytic power, resulting in a data set of 594 patients. The patient characteristics of the pooled data set can be found in Supplementary Table 1.

A representative Probe Selection Region for the genomic span of the short and long NEAT1 isoforms was selected by minimizing the technical variance across the pooled data set. Based on these two Probe Selection Regions, the prognostic performance of NEAT1 short and long isoforms was evaluated using univariable and multivariable odds ratios, and area under the receiver operating characteristics curve for BCR, MET, PCSM and GS>7 endpoints. KM curves were used to perform survival analysis on the Mayo case–cohort patients only^35^, as the nested case–control cohort^58^ was not suitable for KM analysis.

## Additional information

**Accession codes:** Genome-wide data generated in this study have been deposited with The Gene Expression Omnibus (GEO) under accession number GSE43988.

**How to cite this article**: Chakravarty, D. *et al.* The oestrogen receptor alpha-regulated lncRNA NEAT1 is a critical modulator of prostate cancer. *Nat. Commun.* 5:5383 doi: 10.1038/ncomms6383 (2014).

## Supplementary Material

 Supplementary Figures, Tables, Methods and References Supplementary Figures 1-8, Supplementary Tables 1-5, Supplementary Methods and Supplementary References

Supplementary Dataset 1 Information on the patient samples used in this study to identify differentially expressed lncRNAs and RNA statistics.

Supplementary Dataset 2 List of intergenic ERa-regulated lncRNAs (top table): This list reports potential intergenic lncRNAs that are ERa regulated with additional annotation. Columns ERE full and ERE half indicate if the ERa binding site included an ERE motif (full or half, respectively). Analysis of four histone modifications H3K4me3, H3K36me3, H3K9me3, H3K27me3 described in columns 6-9 indicates association of these histone modifications with specific lncRNA. AR regulated indicates insilico analysis of AR binding upstream of specific lncRNA. Benign vs Prostate adenocarcinoma (middle table): The table shows the up-regulated lncRNAs that are sorted by p-values (corrected for multiple hypothesis) between benign and adenocarcinoma (PCa). The highlighted ones are significant at 0.05 levels. Prostate adenocarcinoma vs NEPC (bottom): The table shows the up-regulated lncRNAs that are sorted by p-values (corrected for multiple hypothesis) between adenocarcinoma (PCa) and NEPC. The highlighted ones are significant at 0.05 levels.

Supplementary Dataset 3 Genes upregulated in VCaP ERa cells (log2 fold change > 2) compared to VCaP control cells RNA sequencing of VCaP and VCaP ERa cells was performed and the RPKM values were computed. The expression data was used to identify variation in gene expression between the control and ERa expressing cells. Pairwise comparison between VCaP and VCaP ERa cells revealed 588 genes to be upregulated in VCaP ERa cells (log2 fold change > 2).

Supplementary Dataset 4 Results from Oncomine concepts analysis of ERa gene signature, NEAT1 signature and NEAT1-ERa gene signature.

Supplementary Dataset 5 Genes upregulated in VCaP NEAT1 cells (log2 fold change > 2) compared to VCaP vector control cells RNA sequencing of vector control and VCaP NEAT1 cells was performed and the RPKM values were computed. The table shows the genes that are upregulated in VCaP NEAT1 cells (log2 fold change > 2).
