# Supplementary material for: The oestrogen receptor alpha-regulated lncRNA NEAT1 is a critical modulator of prostate cancer
Source: Nat Commun. 2014 Nov 21;5:5383. doi: 10.1038/ncomms6383 (PMC4241506; doi:10.1038/ncomms6383)
Supplement: Supplementary Figures, Tables, Methods and References — Supplementary Figures 1-8, Supplementary Tables 1-5, Supplementary Methods and Supplementary References [file ncomms6383-s1.pdf]

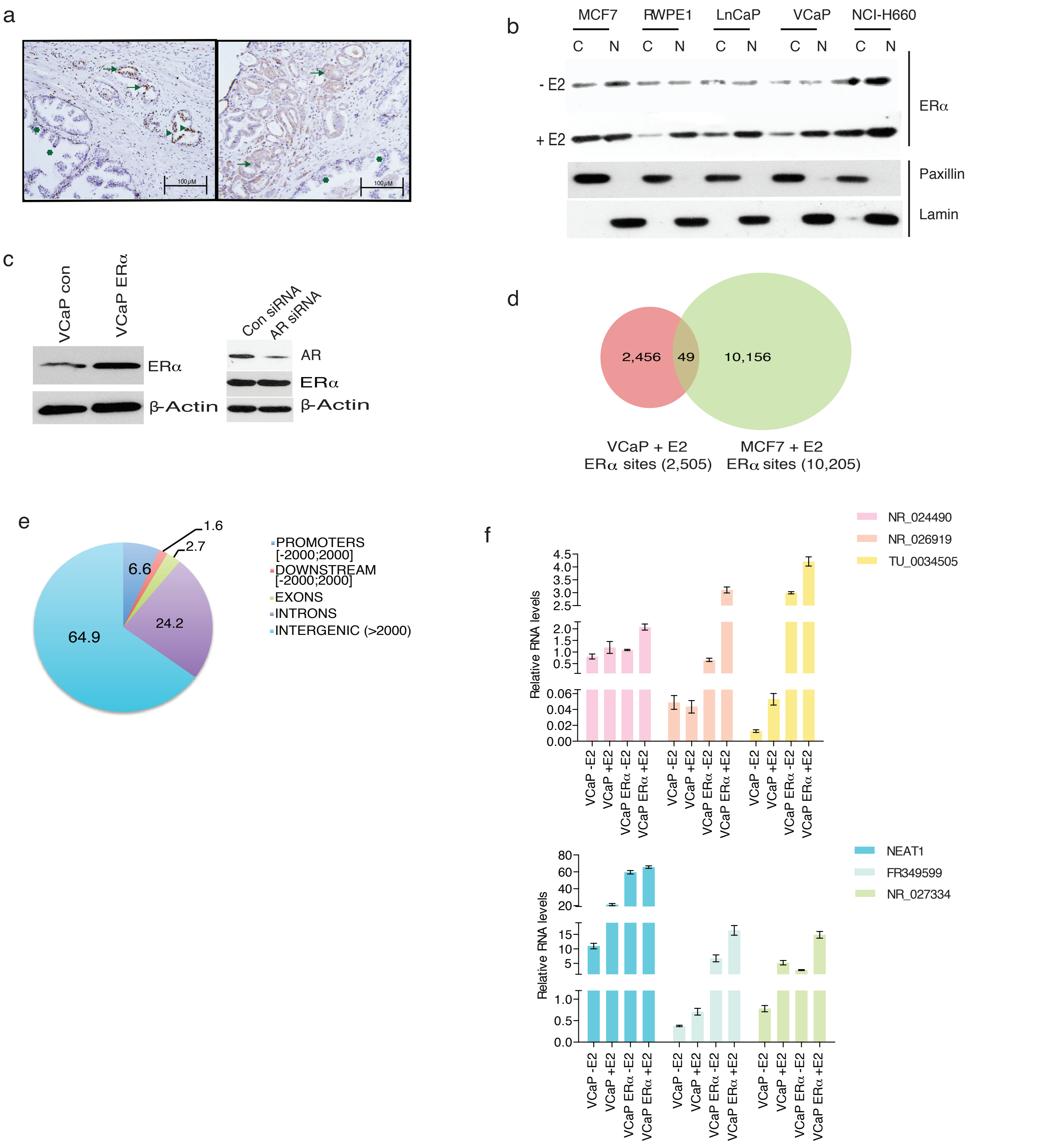

Supplementary Fig. 1

(a) ERα protein expression and localization was evaluated in benign, high grade prostate intraepithelial neoplasia (HGPIN), prostate adenocarcinoma (PCa) and in neuroendocrine prostate cancer (NEPC) cases using immunohistochemistry (IHC) on tissue microarrays. Representative ERα staining in benign prostate, HGPIN and adenocarcinoma is shown on cores; benign prostate (asterisk), HGPIN (arrow head) and PCa (arrow). Focal nuclear staining of ERα was detected in tumor cells and basal cells of adjacent HGPIN (left panel). Focal cytoplasmic staining of ERα was observed in PCa while adjacent benign zones are negative (right panel). (b) ERα expression by western blotting in cytoplasmic and nuclear extracts of MCF7, RWPE1, LnCaP, VCaP and NCI-H660 cells. (c) (left) ERα expression in VCaP control and VCaP ERα expressing cells. (Right) ERα and AR protein levels in VCaP ERα control siRNA and AR siRNA expressing cells (d) Venn diagram demonstrates overlap between ERα peak distributions in VCaP prostate cancer cell line and MCF7 breast cancer cells induced by estrogen. (e) Distribution of ERα binding based on the union of VCaP ERα and NCI-H660 cells ERα binding sites. (f) Expression of ERα regulated lncRNA in VCaP and VCaP ERα cells with and without E2 treatment; Results are expressed as the means±s.d. of three independent experiments. Student's *t*-test was performed for comparisons (relative mRNA levels) between VCaP and VCaP ERα (-E2 condition) and VCaP and VCaP ERα (+E2) conditions.

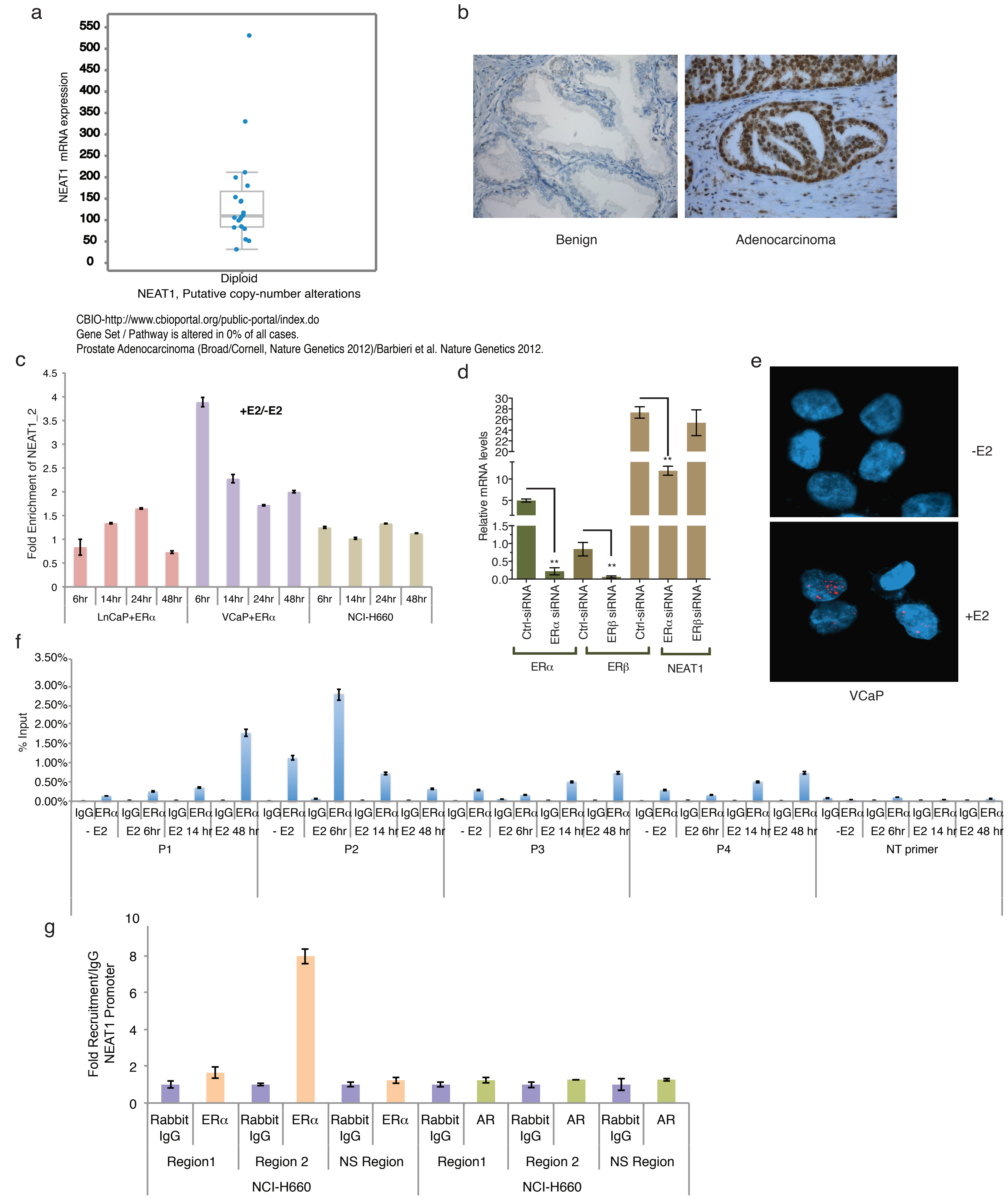

Supplementary Fig. 2
(a) Analysis of NEAT1 using prostate dataset from cBIO portal. (b) Representative image depicting NEAT1 expression in benign and tumor tissue by NEAT1 RNA ISH. (c) qRT-PCR showing NEAT1_2 levels with/without ERα overexpression in different prostate carcinoma cell lines treated with E2 at different time points. Results are expressed as fold enrichment (+E2/-E2). Mean calculated from two independent experiments is shown. Vertical bars represent range of data (d) qRT-PCR analysis for ERα, ERβ and NEAT1 in VCaP cells transfected with control siRNA and either ERα or ERβ siRNA. Results are expressed relative mRNA levels. Mean calculated from two independent experiments is shown. Vertical bars represent range of data (e) Representative image depicting NEAT1 expression by RNA ISH in VCaP cells with and without E2 treatment. (f) Chromatin immunoprecipitation followed by quantitative PCR to study ERα recruitment to NEAT1 promoter in VCaP ERα cells with/without E2 (10nM) treatment. qPCR of ChIP-elute was performed with primers spanning the binding regions identified by ERα ChIP Seq data. Results are expressed as percentage of input calculated from two independent experiments. Vertical bars represent range of data. (g) Recruitment profiles of endogenous ERα to promoter regions of NEAT1 by ChIP followed by quantitative PCR in NCI-H660 cells. Results are expressed as fold recruitment. Mean calculated from two independent experiments is shown. Vertical bars represent range of data.

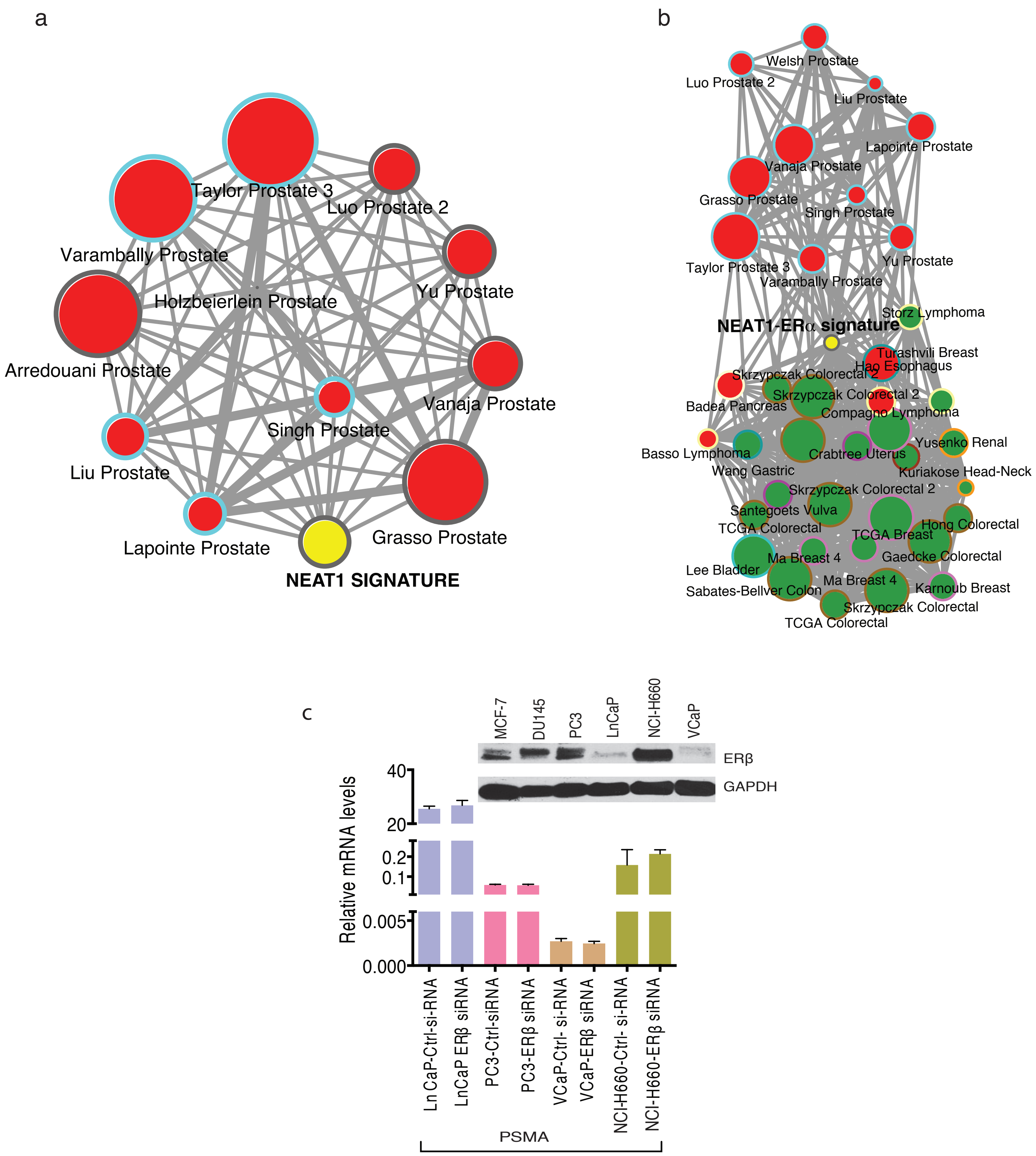

Supplementary Fig. 3
(a) Network representation of NEAT1 signature, derived from genes overexpressed in VCaP NEAT1 cells over vector control cells, across different prostate cancer datasets using Oncomine concept analysis. (b) Network representation of NEAT1-ERα signature, derived from common target genes overexpressed in VCaP ERα (ERα signature) and NEAT1 correlated genes across prostate cancer datasets from Oncomine (NEAT1 signature) cells, across different cancer datasets using Oncomine concept analysis. Each node represent a concept the NEAT1-ERα signature is associated to at a greater than 3-fold odds-ratio. Node size reflect the concept size, i.e. the number of genes in each concept; red and green colors represent correlation with over-or under-expressed genes in the concept respectively; and edge thickness represents the odds-ratio between concepts, ranging from 1.2 to 637. The border color of each node represent the tumor type. (c) inset: Expression of ERβ in different prostate cancer cell lines in comparison to MCF7. Bottom panel: Expression of PSMA in LnCaP, PC3, VCaP and NCI-H660 cells with ERβ knock out. Results are expressed as relative mRNA levels from a representative experiment performed in triplicate (n = 3).

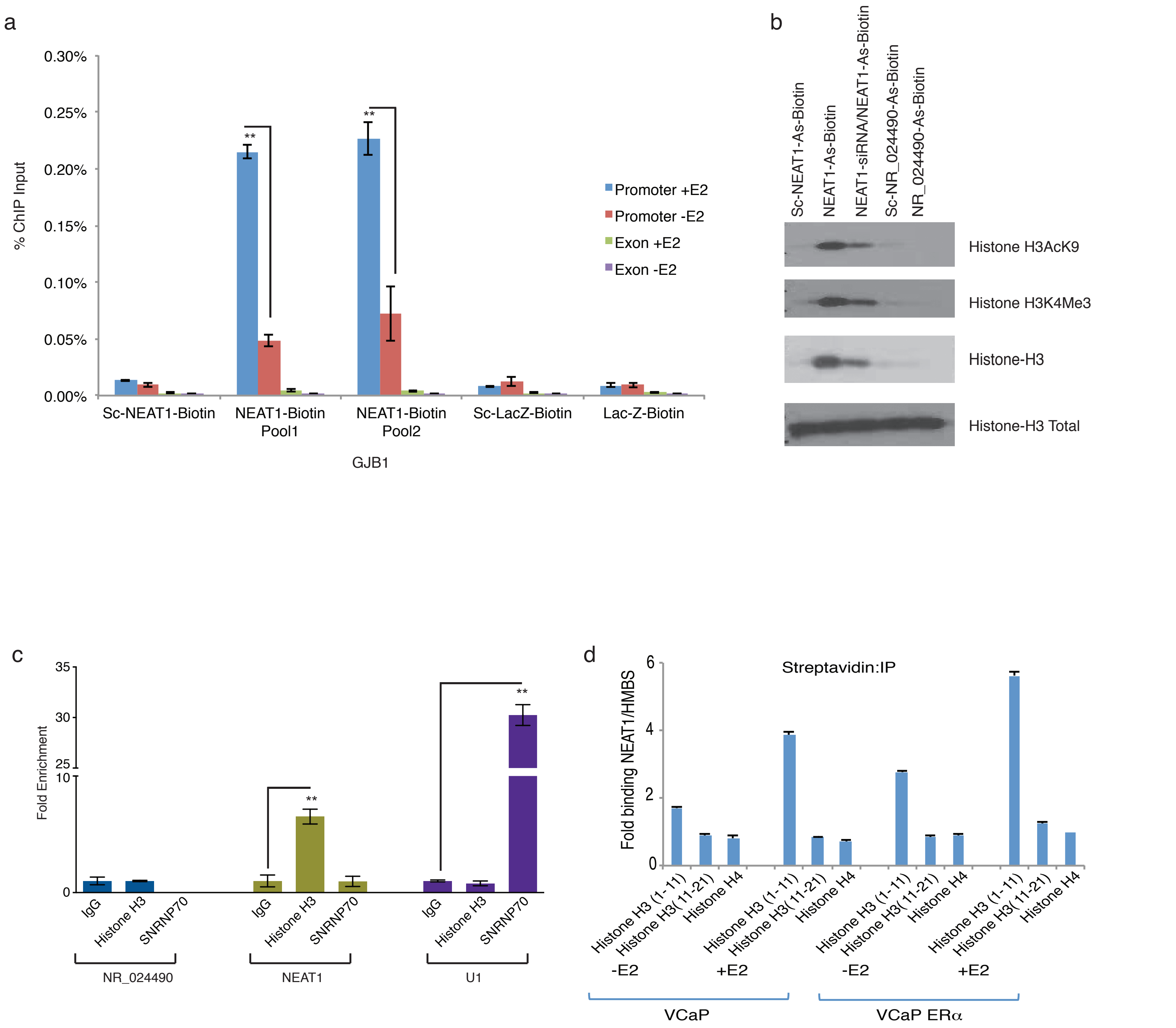

Supplementary Fig. 4
(a) Quantitative analysis of NEAT1 ChIRP in VCaP cells with and without E2 treatment. Recruitment profiles of NEAT1 to GJB1 is shown. Results are expressed as the percentage of input calculated from two independent experiments. Error bars represent the range of data. ** $p<0.01$, Student's *t* test. Results were reproducible between representative experiments. (b) NEAT1 binds to Histone H3 and active histone H3 modifications including, H3AcK9 and H3K4Me3 in NCI-H660 cells. (c) RIP was performed in VCaP ERα cells with anti Histone H3, Normal Rabbit IgG and anti-SNRNP70 as the immunoprecipitating antibody. The figure shows the qPCR data with the purified RNA using primers specific for NR_024490, NEAT1 and U1 snRNA. Mean calculated from two independent experiments is shown. Vertical bars represent range of data (d) NEAT1 specifically recognizes histone H3. Nuclear lysates from VCaP and VCaP ERα cells treated with either vehicle or E2 were used in a streptavidin-biotin pull down assay using biotinylated histone peptides. RNA bound to streptavidin beads was recovered using Trizol and reverse transcribed to obtain cDNA. Level of immunoprecipitated NEAT1 was determined by quantitative PCR. Mean±s.d calculated from three technical replicates is shown. Results were reproducible between representative experiments.

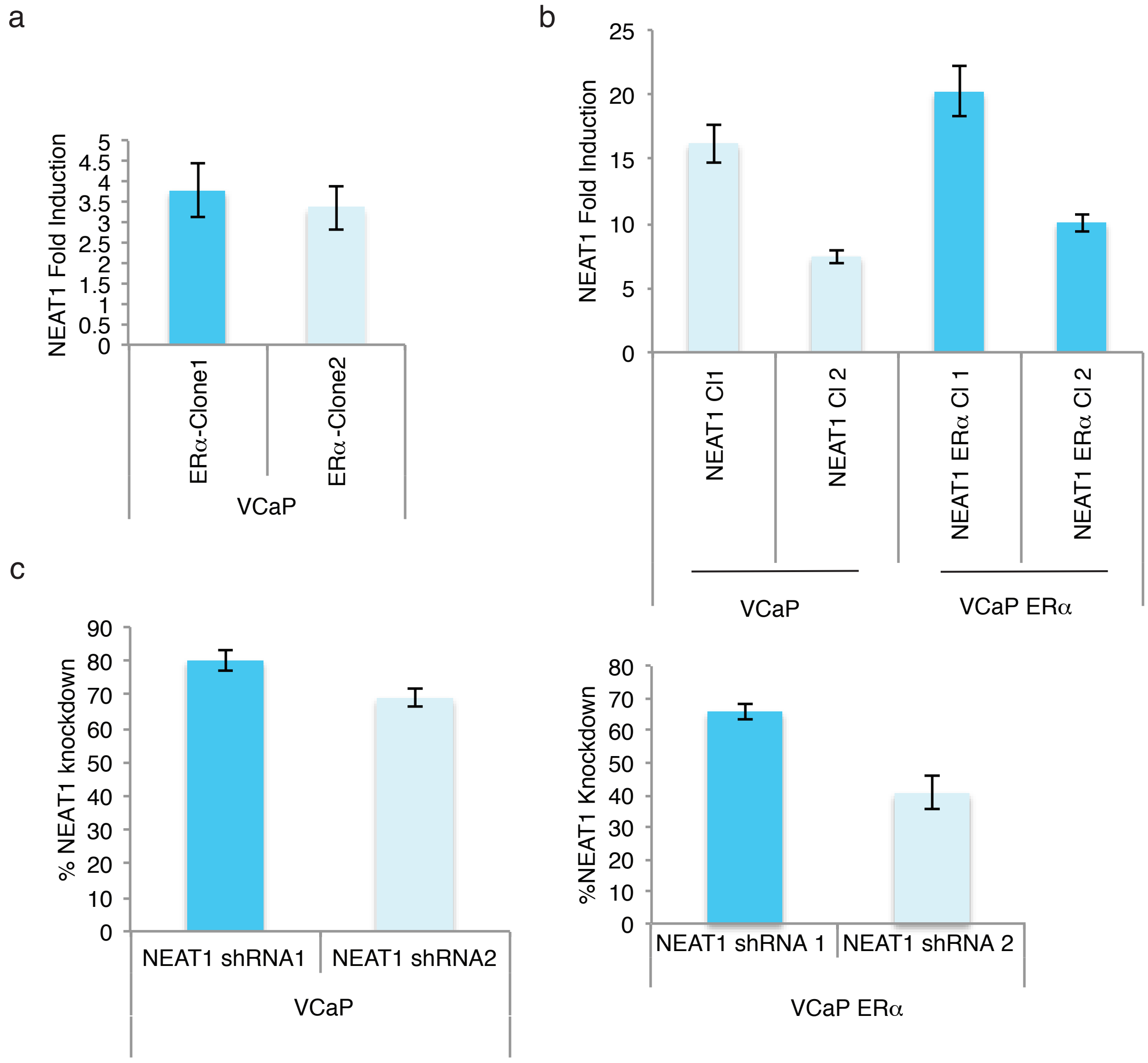

Supplementary Fig. 5
(a) NEAT1 fold induction in VCaP cells overexpressing ERα (b) NEAT1 fold induction in VCaP and VCaP ERα cells overexpressing NEAT1 (c) % knockdown of NEAT1 in VCaP and VCaP ERα cells expressing scrambled shRNA and NEAT1 shRNA. Mean±s.d calculated from three independent experiments is shown. Results were reproducible between representative experiments.

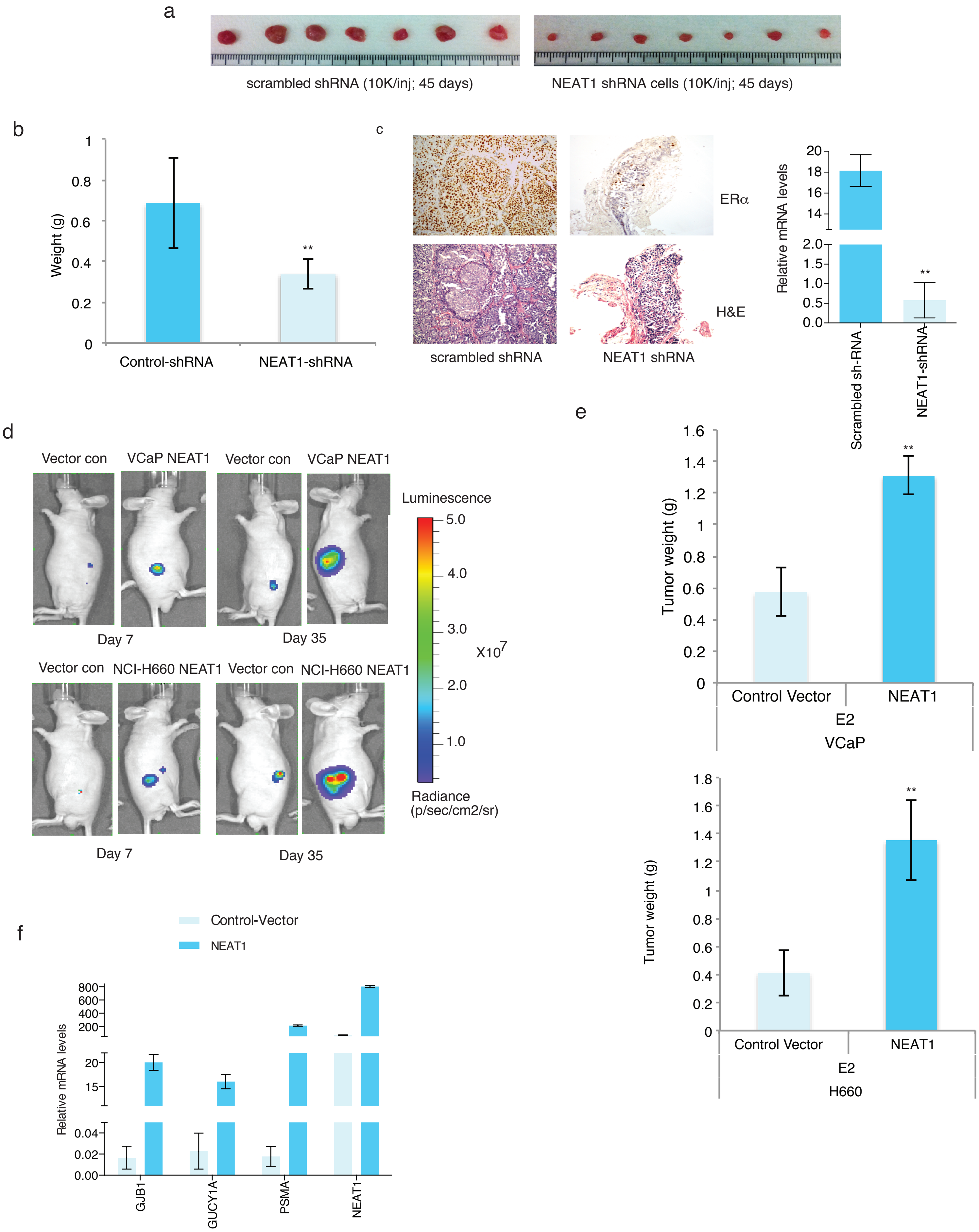

Supplementary Fig. 6

(a) Representative images of tumors derived from mice injected with VCaP ERα cells expressing scrambled shRNA or NEAT1 shRNA. (b) The average tumor weight is shown. Mean±s.d is shown, ** $p < 0.01$, Student's *t*-test. (c) Representative immunohistochemistry on the formalin fixed paraffin embedded tissues for ERα. The lower panel shows the H&E staining for the tumors and on the right we see qRT-PCR data for expression of NEAT1 in the xenografts. Data is presented as mean±s.d from a representative experiment performed in triplicate (n = 3), ** $p < 0.01$. (d) Bioluminescent imaging on Day 7 and Day 35 in the mice injected with VCaP vector control and VCaP NEAT1 cells (top panel) and NCI-H660 vector con and NEAT1 expressing cells (bottom panel). (e) The average weight of tumors from VCaP vector con and NEAT1 expressing group (top panel) and from NCI-H660 vector con and NEAT1 expressing group (bottom panel). Results are expressed as the means±s.d. of three independent experiments. Statistical analysis was performed using Student's *t*-test and ** $p \leq 0.01$. (f) qRT-PCR analysis of relative mRNA levels of 3 ERα NEAT1 signature genes in VCaP vector control and VCaP NEAT1 xenograft tissues; Results are expressed as relative mRNA levels from two independent experiments (cDNA preps). Results were reproducible between representative experiments.

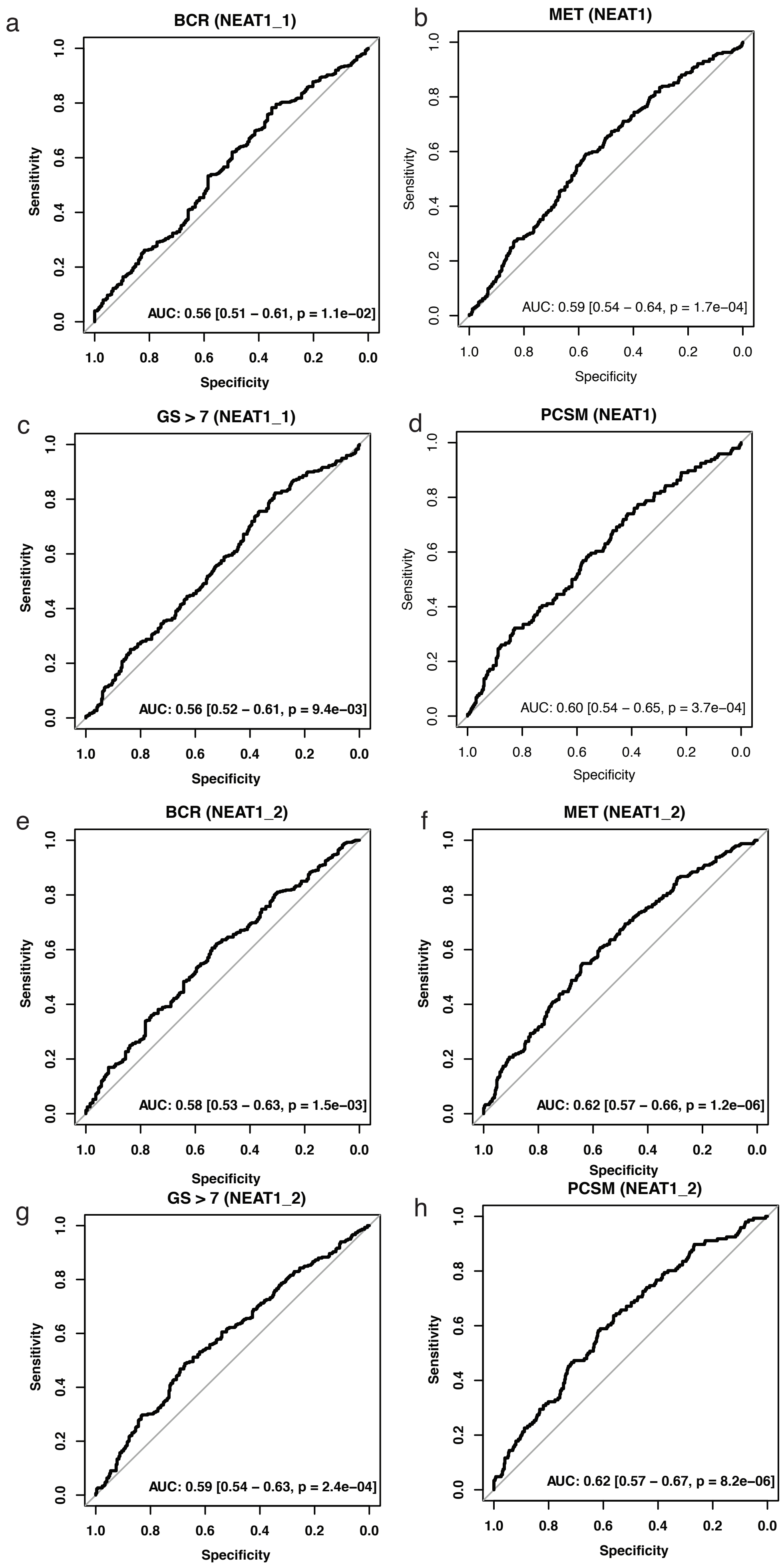

Supplementary Fig. 7
(a-d) AUCs and 95% confidence intervals for the expression of NEAT1_1 transcript predicting BCR (a), MET (b) GS > 7 patient outcomes (c) and PCSM (d) and NEAT1_2 transcript predicting BCR (e), MET (f), GS > 7 patient outcomes (g) and PCSM (h). p-values calculated using the Wilcoxon signed-ranked test indicate that both the long and short forms of NEAT1 are found to be significant prognosticators for each outcome.

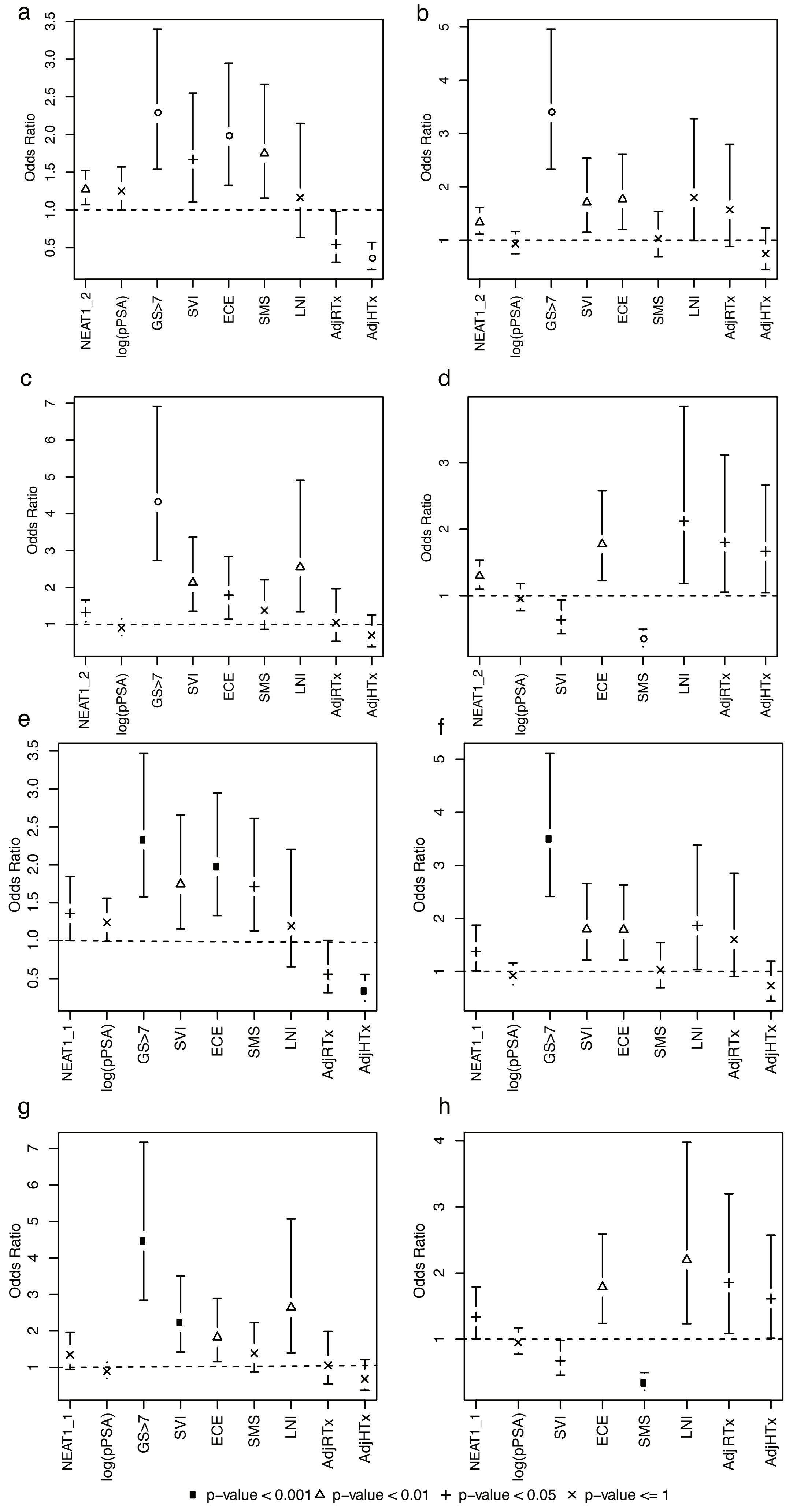

Supplementary Fig. 8

Multivariable odds ratio forest plots for expression of NEAT1_1 and NEAT1_2 transcript.

Forest plots show (a) BCR, (b) MET, (c) PCSM and (d) GS > 7 multivariable odds ratios for NEAT1_2 and (e) BCR, (f) MET, (g) PCSM and (h) GS > 7 multivariable odds ratios for NEAT1_1 adjusted for clinicopathologic factors and adjuvant treatment (n = 594). NEAT1 is found to contribute significant independent information for the prognostication of each endpoint.

Supplementary Table 1: Patient characteristics for the pooled Mayo nested case-control and Mayo case-cohort datasets.

	%
BCR	68
MET	41
PCSM	25
GS > 7	50
pT3+	68
LNI	18
SMS	62
SVI	42
ECE	53
pPSA > 20	29
AdjHTx	33
AdjRTx	12

Supplementary Table 1: Patient characteristics for the pooled Mayo nested case-control and Mayo case-cohort datasets.
Biochemical recurrence (BCR), metastatic recurrence (MET), prostate cancer specific mortality (PCSM), Gleason score (GS) > 7, pathological tumor stage 3 or greater (pT3+), Lymph Node Invasion (LNI), Surgical Margin Status (SMS) positive, Seminal Vesicle Invasion (SVI), Extra Capsular Extension (ECE), preoperative PSA (pPSA), adjuvant hormone therapy, and adjuvant radiation therapy are shown.

Supplementary Table 2: Clinical information of the patient samples used to derive correlation between NEAT1 and ERα expresssion

Sample ID	Age	Diagnosis	Sample Type	Site	Overall Gleason Score	Treatment
10680_B	72	Benign	Prostatectomy	Prostate	NA	NA
15489_B	73	Benign	Prostatectomy	Prostate	NA	NA
16748_B	67	Benign	Prostatectomy	Prostate	NA	NA
20870_B	62	Benign	Prostatectomy	Prostate	NA	NA
21090_B	52	Benign	Prostatectomy	Prostate	NA	NA
21017_B	72	Benign	Prostatectomy	Prostate	NA	NA
21070_B	79	Benign	Prostatectomy	Prostate	NA	NA
21811_B	77	Benign	Prostatectomy	Prostate	NA	NA
PM0_B	55	Benign	Prostatectomy	Prostate	NA	NA
15489_T	73	Prostate adenocarcinoma	Prostatectomy	Prostate	7	Hormone Naïve
15508_T	70	Prostate adenocarcinoma	Prostatectomy	Prostate	9	Hormone Naïve
15969_T	64	Prostate adenocarcinoma	Prostatectomy	Prostate	7	Hormone Naïve
16288_T	67.6	Prostate adenocarcinoma	Prostatectomy	Prostate	7	Hormone Naïve
16308_T	67	Prostate adenocarcinoma	Prostatectomy	Prostate	9	Hormone Naïve
18208_T	49	Prostate adenocarcinoma	Prostatectomy	Prostate	7	Hormone Naïve
20730_T	49	Prostate adenocarcinoma	Prostatectomy	Prostate	7	Hormone Naïve
PM0	55	Metastatic prostate Cancer	Rapid Autopsy	Liver	NA	Chemotherapy
PM59	77	Metastatic prostate Cancer	Biopsy	Liver	NA	ADT, Chemotherapy
PM90	62	Metastatic prostate Cancer	Biopsy	Liver	NA	ADT, Chemotherapy
PM159	63	Metastatic prostate Cancer	Rapid Autopsy	Lymph node	NA	ADT, Chemotherapy
PM89	89	Metastatic prostate Cancer	Biopsy	Lymph node	NA	ADT, Chemotherapy
PM169	66	Metastatic prostate Cancer	Biopsy	Bone	NA	ADT
PM195	85	Metastatic prostate Cancer	Transurethral Resection	Prostate	NA	ADT, XRT, Chemotherapy

ADT-Androgen Deprivation Therapy
XRT-Radiation Therapy

Supplementary Table 3: Sequence of siRNAs used in this study

**Target**	**Seq.**
siNEAT1	UGGUAAUGGUGGAGGAAGAUU
siNEAT1	GUGAGAAGUUGCUUAGAAAUU
siNEAT1	GGAGGAGUCAGGAGGAAUAUU
siNEAT1_2	CCAAAUAGGCUUACAGAUAUU
siNEAT1_2	AGAGAGAAGUUGUGGAGAAUU
ESR1 ON-TARGETplus	GAUCAAACGCUCUAAGAAG<br>GAAUGUGCCUGGCUAGAGA<br>GAUGAAAGGUGGGAUACGA<br>GCCAGCAGGUGCCCUACUA
ESR1 Accell	UAUUCAUGUUAAGAUACUA<br>GGAAGGUUUUACAUUAUUC<br>GCCUGGUGAUUAUUCAUUU<br>CCGUAAUGAUUCUAUAAUG
ESR2 ON-TARGETplus	GGAAAUGCGUAGAAGGAAU<br>UUCAAGGUUUCGAGAGUUA<br>GCACGGCUCCAUAUACAUA<br>GAACCCACAGUCUCAGUGA
AR ON-TARGETplus	GAGCGUGGACUUUCCGGAA<br>UCAAGGAACUCGAUCGUAU<br>CGAGAGAGCUGCAUCAGUU<br>CAGAAAUGAUUGCACUAUU
piLenti-NEAT1 siRNA-GFP	TCATGGACCGTGGTTTGTTACTATAGTGT<br>GTTCTTAGCCTGATGAAATAACTTGGGGC<br>GTGAGAAGTTGCTTAGAAACTTTCC<br>CTGGTATGTTGCTCTGTATGGTAAG
iLenti-si-scrambled	CAACCCGCTCCAAGGAATCG

Supplementary Table 4: Primer sequences used in this study

**Gene**	**Sequence (5' --> 3')**
ERbeta	ERbeta F: 5’ – CTGCTGAGTCTTGGCTGTCA – 3’
	ERbeta R: 5’ – GGATGTGGCAAAGCACTACA – 3’
AR	AR F: 5’ – CCAGGGACCATGTTTTGCC – 3’
	AR R: 5’ – CGAAGACGACAAGATGGACAA – 3’
	AR F: 5’ – CGAAGACGACAAGATGGACAA – 3’
AR	AR R: 5’ – CCAGGGACCATGTTTTGCC – 3’
	STAT3 F: 5’ – ACCAGCAGTATAGCCGCTTC – 3’
STAT3	STAT3 R: 5’ – GCCACAATCCGGGCAATCT – 3’
	TMPRSS2 F: 5’ – GGACAGTGTGCACCTCAAAGAC – 3’
TMPRSS2	TMPRSS2 R: 5’ – TCCCACGAGGAAGGTCCC – 3’
	ESR1_Lisa F: 5’ – GCTGTTCTTCTTAGAGCGTTTGA – 3’
ESR1 (ERα)	ESR1_Lisa R: 5’ – GAAAGGTGGGATACGAAAAGACC – 3’
	NEAT1_2 F: 5’ – TTTGTGCTTGGAACCTTGCT – 3’
NEAT 1	NEAT1_2 R: 5’ – TCAACGCCCCAAGTTATTTC – 3’
	NEAT1v2 F: 5' – TCTCCATTTCCCCATCTGAG – 3'
NEAT 1_2	NEAT1v2 R: 5' – CAGCCACAGAAAAGGGAGAG – 3'
	HMBS F: 5’ – AGCTTGCTCGCATACAGACG – 3’
HMBS	HMBS R: 5’ – AGCTCCTTGGTAAACAGGCTT – 3’
	HPRT F: 5’ – GAAGTTTTGTTCTGTCCTGGAA – 3’
HPRT	HPRT R: 5’ – GGGAACTGCTGACAAAGATTC – 3’
	EHF F: 5’ – GGACCAAGTACCAGGTGTGG – 3’
EHF	EHF R: 5’ – GCTGCAAGTTGCTGTAGAGG – 3’
	PPM1E F: 5’ – CCCGAACCTGAACTGGTAGA – 3’
PPM1E	PPM1E R: 5’ – CTCCTCCTCCTCACCCTCA – 3’
	SIM2 F: 5’ – GGCTACTTGAAGATCAGGCAGT – 3’
SIM2	SIM2 R: 5’ – TCCAGGAATATCAGCTTCAGG – 3’
	ADRB1 F: 5’ – CGAGACCCTGTGTGTCATTG – 3’
ADRB1	ADRB1 R: 5’ – ACTTGGGGTCGTTGTAGCAG – 3’
	HPN F: 5’ – GTCCCGATGGCGAGTGTT – 3’
HPN	HPN R: 5’ – AGGGCAATATCGTTGCTGTT – 3’
	PLA2G7 F: 5’ – TCATCAGCATGGGTCAACAAAA – 3’
PLA2G7	PLA2G7 R: 5’ – CCAAAGGGTGTCAAGGCGAT – 3’
	PDLIM3 F: 5’ – CCAACCTGTGTCCTGGAGAT – 3’
PDLIM3	PDLIM3 R: 5’ – TTGAGACACAGCTGGTGAGC – 3’
	AMACR F: 5’ – TCCGGTTCTGACTTTTGAGG – 3’
AMACR	AMACR R: 5’ – CGGCTGAATCCAAATTCTTC – 3’
	RTN1 F: 5’ – GACCTGTTGTATTGGCGGGAC – 3’
RTN1	RTN1 R: 5’ – GTAGATGCGGAAACTGATGGTG – 3’
	GJB1 F: 5’ – GAAAATGCTACGGCTTGAGG – 3’
GJB1	GJB1 R: 5’ – TAGACGTCGCACTTGACCAG – 3’
	SPDEF F: 5’ – CAGTGCCCGGTCATTGACA – 3’
SPDEF	SPDEF R: 5’ – CAGCCGGTATTGGTGCTCT – 3’
	ARHGAP6 F: 5’ – TTTGGAATGCCCTTATCCCAAG – 3’
ARHGAP6	ARHGAP6 R: 5’ – TCTGCTCGTCCCTCTGCAA – 3’
	CHGB F: 5’ – GAGCCTCTATCCCTCCGACAG – 3’
CHGB	CHGB R: 5’ – CCCTCGCTCCCCTTTTTGA – 3’
	PRAC F: 5’ – CTAGCCTAAGGCGTGCAAAC – 3’
PRAC	PRAC R: 5’ – CCGGTCCTTGATCTGAGAAA – 3’
	ACPP F: 5’ – GCTTCCCCTCTACAGGCTTT – 3’
ACPP	ACPP R: 5’ – GCAGAAACAAGAAGCCAAGG – 3’
	PSMA F: 5’ – CATAGTGCTCCCTTTTGATTGTC – 3’
PSMA/FOLH1	PSMA R: 5’ – CTCTCACTGAACTTGGAAGCAAT – 3’
	B3GAT1 F: 5’ – CCTGGCGTGGTCTACTTCG – 3’
B3GAT1	B3GAT1 R: 5’ – GCAGGTTGACGGCAAATCC – 3’

ALDH1A1	ALDH1A1 F: 5’ – GAAGAAAGAAGGGGCCAAAC – 3’<br>ALDH1A1 R: 5’ – CAATGCGCATCTCATCTGTAA – 3’
TRPM8	TRPM8 F: 5’ – GAAACCTGTCGACAAGCACA – 3’<br>TRPM8 R: 5’ – GAGGACAAAGACCAGCGAGT – 3’
GUCY1A3	GUCY1A3 F: 5’ – GATTGCAGCGAGTTTGTGAA – 3’<br>GUCY1A3 R: 5’ – ATCAGCCTTCTGATGCCATT – 3’
ERG	ERG F: 5’ – TTGTGAGTGAGGACCAGTCG – 3’<br>ERG R: 5’ – CCTGGCTAGGGTTACATTCC – 3’
NPY	NPY F: 5’ – GCTAGGTAACAAGCGACTGG – 3’<br>NPY R: 5’ – CTGCCTGGTGATGAGGTTG – 3’
KLK2	KLK2 F: 5’ – CACAACCTGTTTGAGCCTGA – 3’<br>KLK2 R: 5’ – CCCAGGACCTTCACAACATC – 3’
LDHB	LDHB F: 5’ – TGGTATGGCGTGTGCTATCAG – 3’<br>LDHB R: 5’ – TTGGCGGTCACAGAATAATCTTT – 3’
CRISP3	CRISP3 F: 5’ – AAAACCCTGGATTGAAAGTGAA – 3’<br>CRISP3 R: 5’ – AGCCAGATGATTGGGAAGAA – 3’
ACPP	ACPP F: 5’ – GCTTCCCCTCTACAGGCTTT – 3’<br>ACPP R: 5’ – GCAGAAACAAGAAGCCAAGG – 3’
STEAP2	STEAP2 F: 5’ – CAAGCGCGACAACAGGTTATT – 3’<br>STEAP2 R: 5’ – CAGAGAGTAAAGAGTCGTAGGGG – 3’
PRAC	PRAC F: 5’ – AAGGACCGGCCCATCTTACTA – 3’<br>PRAC R: 5’ – CCTGGTCTCGCCCAGTAGA – 3’
CLDN8	CLDN8 F: 5’ – CAACCCATGCCTTAGAAATCGC – 3’<br>CLDN8 R: 5’ – TCACGCAATTCATCCACAGTC – 3’
STEAP1	STEAP1 F: 5’ – CCCTTCTACTGGGCACAATACA – 3’<br>STEAP1 R: 5’ – GCATGGCAGGAATAGTATGCTTT – 3’
KLK4	KLK4 F: 5’ – TCAGCCGCACACTGTTTCC – 3’<br>KLK4 R: 5’ – GGGTCTGTTGTACTCTGGGTG – 3’
TMPRSS2	TMPRSS2 F: 5’ – GGACAGTGTGCACCTCAAAGAC – 3’<br>TMPRSS2 R: 5’ – TCCCACGAGGAAGGTCCC – 3’
NR_024490	NR_024490 F: 5’ – TTCCTGTGCTAACCTTCGTG – 3’<br>NR_024490 R: 5’ – GGTACCTTCACAGCGTCTCC – 3’
FR349599	FR349599 F: 5’ – CCTGGCTTCACCGCATTC – 3’<br>FR349599 R: 5’ – AGGGTGGTCCTGAGAGTGGT – 3’
FR330851	FR330851 F: 5’ – TCATTGCATGGTTGGAAATG – 3’<br>FR330851 R: 5’ – CAACTTTCTGCCTTCAACTGC – 3’
NR_026919	NR_026919 F: 5’ – TGGGCTCTGACCTCTGTACC – 3’<br>NR_026919 R: 5’ – CTGATAAATGTCCCCGTGCT – 3’
NR_027334	NR_027334 F: 5’ – CAGCCCTACCTGTCCTGATG – 3’<br>NR_027334 R: 5’ – ACTGAGCCTCCAACTCCAGA – 3’
U1 snRNA	F: 5'-GGG AGA TAC CAT GAT CAC GAA GGT-3'<br>R: 5'-CCA CAA ATT ATG CAG TCG AGT TTC CC-3'

Primers used in ChIP Assay

ESR1_P_1	ESR1_P_1 F: 5’ – GTGAAACCCCGTCTCTACCA – 3’<br>ESR1_P_1 R: 5’ – TGGGAAGACAACCTGTAGGG – 3’
ESR1_P_2	ESR1_P_2 F: 5’ – AGCAGGCCAATCTAGAAGCA – 3’<br>ESR1_P_2 R: 5’ – CAGCAATGGAAGGACAGAACT – 3’
ESR1_P_3	ESR1_P_3 F: 5’ – AATGGAATGGACTCGAATGG – 3’<br>ESR1_P_3 R: 5’ – ATTGCAATCCATTTCTTTCG – 3’
ESR1_P_4	ESR1_P_4 F: 5’ – TGGAATCGAATCACATGGAA – 3’<br>ESR1_P_4 R: 5’ – CCATTCCACACCTCTCCAGT – 3’

ESR1_P_5	ESR1_P_5 F: 5’ – GGCATCGAATGAAATGGAAT – 3’<br>ESR1_P_5 R: 5’ – TTCAAATAAATTCCGCTCGTG – 3’
ESR1_P_6	ESR1_P_6 F: 5’ – CCTCGAGCGGAATTTATTTG – 3’<br>ESR1_P_6 R: 5’ – TCGATTTCGTTCCCTTTGAT – 3’
ESR1_P_7	ESR1_P_7 F: 5’ – TGGAGTGTAACGGGATGATG – 3’<br>ESR1_P_7 R: 5’ – TCTCCATTCGAGTCCATTCC – 3’
ESR1_P_8	ESR1_P_8 F: 5’ – GAATGGAATGGAAGGCAATG – 3’<br>ESR1_P_8 R: 5’ – CAAGCCACTTCAATCCCCTA – 3’
ESR1_P_9	ESR1_P_9 F: 5’ – AATAGAATGGAACGGCATCG – 3’<br>ESR1_P_9 R: 5’ – CCATTCGAATCCAATACACTCA – 3’
ESR1_P_10	ESR1_P_10 F: 5’ – TGAAATGGAGCGCATTAGAA – 3’<br>ESR1_P_10 R: 5’ – TGTCCATTCCATTTGATTGC – 3’
ESR1_P_11	ESR1_P_11 F: 5’ – GCTCCAGCATTCTGACCTCT – 3’<br>ESR1_P_11 R: 5’ – CAGGAGCAACTCTGGGTCTC – 3’
PSMA/FOLH1-P_1	PSMA-P_1 F: 5’ – TGCACGGCCTCTCTCACGGA – 3’<br>PSMA-P_1 R: 5’ – GGCTATGTCTGGCTACTGTCTTA – 3’
PSMA/FOLH1-P_2	PSMA-P_2 F: 5’ – TCGAGAAATCGAGACCATCC – 3’<br>PSMA-P_2 R: 5’ – ACTCTGTCACCAGGCTCCAG – 3’
PSMA/FOLH1_coding	PSMA-F:5'-GGGTCTTGATGCTGTGGTCT-3'<br>PSMA-R:5'-ATCTTTCCCCTATGCCTGGT-3'
B3GAT1-P_1	B3GAT1-P_1 F: 5’ – ACCCGAGCTGCTAATGGTC – 3’<br>B3GAT1-P_1 R: 5’ – GGTGGGGAGGTGATACCAAT – 3’
B3GAT1-P_2	B3GAT1-P_2 F: 5’ – CCCCAGATCTCGGCTCTC – 3’<br>B3GAT1-P_2 R: 5’ – CTTTGAGAGAGGCTGCCAAG – 3’
B3GAT1-P_3	B3GAT1-P_3 F: 5’ – CACTGGTAGGGTGCCTCTGT – 3’<br>B3GAT1-P_3 R: 5’ – CCCGAATACAGGTGTCCAAG – 3’
B3GAT1-P_4	B3GAT1-P_4 F: 5’ – TCTAGGGTCTCCCTCCTTCC – 3’<br>B3GAT1-P_4 R: 5’ – GCAGGAAAGCTGATGGAAAA – 3’
GJB1-P_1	GJB1-P_1 F: 5’ – TCCAGCTCAGTTTTGCCTTT – 3’<br>GJB1-P_1 R: 5’ – GGGAGGAGGAACAGTGTGAG – 3’
GJB1-P_2	GJB1-P_2 F: 5’ – CGGCCCTGTCATACAGTTTT – 3’<br>GJB1-P_2 R: 5’ – ATTGTCACCCACCCATGTTT – 3’
GJB1_coding	GJB1-F: 5'-GAAAATGCTACGGCTTGAGG-3'<br>GJB1-F: 5'-TAGACGTCGCACTTGACCAG-3'
NEAT1_P_1	NEAT1_P_2 F: 5’ – GGCAGTGACCCTGACAAGTT – 3’<br>NEAT1_P_2 R: 5’ – TCTGAAGGAGCCTTTTCTGC – 3’
NEAT1_P_2	NEAT1_P_3 F: 5’ – CCATCTGACCTTGGCACTTT – 3’<br>NEAT1_P_3 R: 5’ – CAACCCACCTTGGTCTGACT – 3’
NEAT1_P_3	NEAT1_P_4 F: 5’ – GGGAACTCCCTTCCTCAGTC – 3’<br>NEAT1_P_4 R: 5’ – CAGCCTCCTGACCACAACTC – 3’
NEAT1_P_4	NEAT1_P_1 F: 5’ – AATTTTCCAGATGTCCTGCC – 3’<br>NEAT1_P_1 R: 5’ – AACAGTGCTTTTTGGGATCG – 3’
NT-NEAT1_P1	ER_P_NT-NEAT1_1 F: 5’ – TCATTGGTCATGGCTTAGCA – 3’<br>ER_P_NT-NEAT1_1 R: 5’ – AGCCATTTTGTCTCCTGCAC – 3’
NT-NEAT1_P2	ER_P_NT-NEAT1_2 F: 5’ – ACTATTTCAACCGCCACGAG – 3’<br>ER_P_NT-NEAT1_2 R: 5’ – TGCCTGAGGGCTCTAGTCAT – 3’
U1 snRNA	FOR: GGG AGA TAC CAT GAT CAC GAA GGT<br>REV: CCA CAA ATT ATG CAG TCG AGT TTC CC

Supplementary Table 5: Biotin TEG antisense probes

Biotin TEG probes:

Sequence Name	Sequence (5'-3')
NEAT1_scr1	gagaacatcgaattagcgtc
NEAT1_scr2	gactatcagacgcctaagat
NEAT1_scr3	ggtacgtaacgaggaagcga
NR_024490_1	ttaaattcttggcgctgtct
NR_024490_2	aagtcttccataataccctt
NR_024490_3	actcagtccaagtcattact
LacZ_1	tcacgacgttgtaaaacgac
LacZ_2	gctgatttgtgtagtcggtt
LacZ_3	tttaccttgtggagcgacat
HOTAIR_1	attttaggaggcccaaacag
HOTAIR_2	tgttcactctggcaagagat
HOTAIR_3	actttgcccagattaaacgt
NEAT1_185	tgcggatattttccatgcag
NEAT1_488	agcccttggtctggaaaaaa
NEAT1_800	aagcgttggtcaatgttgtc
NEAT1_1161	tcgccatgaggaacactata
NEAT1_1516	tgcagcatctgaaaaccttt
NEAT1_1829	cacaacacaatgacaccctt
NEAT1_2195	actgtatctctaaccaaccc
NEAT1_2555	ccaggaggaagctggtaaag
NEAT1_2939	gatgtgtttctaaggcacga
NEAT1_3325	ccattggtattactttacca
NEAT1_3691	ttatttgtgctgtaaagggg

Supplementary Methods

Tissues:

The prostate cancer tissue microarrays include 64 benign, 16 high grade PIN, 292 adenocarcinoma and 42 neuroendocrine prostate cancer specimens. Tissues were obtained from radical prostectomy series at the Weill Cornell Medical College. All samples were collected with informed consent of the patients and under an Institutional Review Board approved protocol. Hematoxilin and eosin (H&E) stained FFPE and frozen sections were reviewed by study pathologist (M.A.R./J.M) to identify regions of high density cancer foci, or high grade prostate intraepithelial neoplasia or lack of tumor.

Immunohistochemical staining of Tissue microarrays:

Sections were deparaffinized with xylene and rehydrated using graded ethanol. The hydrated sections were boiled for 10 minutes in 0.01mol/L citrate buffer and cooled for 30 minutes at room temperature to expose antigenic epitopes. Tissue slides were blocked for 30 min at room temperature with 2% normal goat serum and 1% BSA in PBS for 30 minutes. Tissue cores were incubated with rabbit monoclonal antihuman-ERα antibody (RM-9101, Thermo scientific) at a dilution of 1:100 and incubated overnight at room temperature. The sections were washed thrice with 0.05% Tween in PBS for 10 minutes, incubated with secondary antibody for 1 hour, washed thrice with 0.05% Tween in PBS for 10 minutes, developed with 3,3′-diaminobenzidine-H_2O_2, (DAB) and then counterstained with Mayer's hematoxylin. Negative controls were done by replacing the primary antibody with control rabbit IgG or peptide-absorbed ERα antibody. For each run a positive tissue control (breast cancer) was included. Subjective evaluation of ERα protein expression was scored as positive or negative by pathologists

for the study. The same protocol was also followed for immunohistochemical staining on the xenograft tissue sections for ERα.

RNA isolation, cDNA synthesis and PCR experiments:

Total RNA was isolated from frozen prostate tissue samples (for qPCR) and cell lines (for transcriptome sequencing and qPCR) using Trizol (Invitrogen) with DNase I digestion according to manufacturer's instructions. RNA integrity was verified on an Agilent Bioanalyzer 2100 (Agilent Technologies). cDNA was synthesized from total RNA using Superscript III (Invitrogen) and random primers (Invitrogen).

Quantitative RT-PCR was performed using Power SYBR Green Mastermix (Applied Biosystems) on an Applied Biosystems 7900 Fast Real Time PCR machine. All primers were designed using Primer 3 and synthesized by Integrated DNA Technologies. Primer sequences are provided in **Supplementary Table 5**.

Statistical analysis for quantitative RT-PCR:

For quantitative real time PCR, we subtracted the mean CT value from each gene "*g*" to the mean control value (HMBS) to compute the Delta CT value: $\Delta CT_g = \overline{CT_g} - \overline{CT_{HMBS}}$. The standard deviation of this value was calculated as the square root of the sum of the squares of the standard deviations: $SD_{\Delta CT} = \sqrt[2]{SD_g^2 + SD_{HMBS}^2}$. We then computed $\Delta\Delta CT_g = 2^{-(\Delta CT_g)}$ for each gene and condition and considered each value to be within this range $\left[2^{-(\Delta CT_g - |SD_{\Delta CT}|)}, 2^{-(\Delta CT_g + |SD_{\Delta CT}|)}\right]$. Finally we computed the fold changed between condition C1 and C2, e.g. C1=with E2 and C2=without E2 as: $FC = \frac{\Delta\Delta CT_g^{C1}}{\Delta\Delta CT_g^{C2}}$. The standard deviation of this value was computed according to the rules

of propagation of error, i.e. $\frac{SD_{FC}}{FC} = \sqrt[2]{\left(\frac{SD_{C1}}{\Delta\Delta CT_g^{C1}}\right)^2 + \left(\frac{SD_{C1}}{\Delta\Delta CT_g^{C2}}\right)^2}$; where SD_{Ci} is the range of $\Delta\Delta CT_g^i$ for condition *i*.

Western Blot:

Cells were fractionated and lysed as described previously. Standard protocols were followed for western blotting[1]. PVDF membranes (GE Healthcare) were used for western blotting and immunoblotted using specific antibodies.

Antibodies and the dilutions used: Anti AR (06-680, Millipore, 1:1000), Anti ERα (AC-066-100, diagenode, 1:1000), Anti Ace-H3 (06-599, Millipore, 1:1000), Anti H3K4me3 (ab8580, Abcam, 1:1000), Anti GAPDH (AB2302, Millipore, 1:10000), Anti Actin (MAB1501, Millipore, 1:1000), Anti Lamin B1 (ab28129, Abcam, 1:1000), Anti Histone H3 (06-755, Millipore, 1:1000), Anti Paxillin (05-417, Millipore, 1:1000), Anti ERβ (ab288, Abcam, 1:1000).

RNA imunoprecipitation (RIP assay):

RIP assays were performed using Milipore EZ-Magna RIP kit (17-701) according to the manufacturer's instruction. Briefly, cells were lysed in RIP lysis buffer, followed by immunoprecipitation with antibody to Histone H3 (ab1791, Abcam, 5μg), Anti-SNRNP70 (CS203216, Millipore, 5μg) and negative control Normal Rabbit IgG (PP64B, Millipore) with protein A/G magnetic beads. The magnetic bead bound complexes were washed to get rid of unbound materials and the RNA was extracted and subsequently analyzed by qRT-PCR.

Peptide pull-down assay:

Nuclear lysates from VCaP and VCaP ERα cells treated with either vehicle or E2 were used in a streptavidin-biotin pull down assay using biotinylated histone peptides[2]. RNA bound to streptavidin beads was recovered using Trizol and reverse transcribed to obtain cDNA. Levels of immunoprecipitated NEAT1 were determined by quantitative PCR.

RNA sequencing:

Standard poly-A selected RNA sequencing was done for VCaP, VCaP ERα expressing cells as well as for VCaP cells overexpressing empty vector and VCaP NEAT1 over-expressing cell lines using Illumina TruSeq RNA-seq protocol.

Reads were aligned to the reference genome NCBI36/hg18 without the minor haplotypes and the minor sequences using STAR aligner[3]. Reads mapped to the mitochondrial genome were removed. The expression of each gene (UCSC knownGenes) was computed using mrfQuantifier, part of RSEQtools[4]. The resulting expression data was used to identify variation in gene expression between VCaP and VCaP ERα cells and also between the vector control and NEAT1 expressing cells. We computed the ratio between VCaP and VCaP ERα and control cells and VCaP NEAT1, after adding 1, and selected those genes with a log2-fold change greater than 2. Results are reported in **Supplementary dataset 3** and **Supplementary dataset 4.**

ChIP sequencing:

ERα ChIP followed by sequencing was performed in VCaP, VCaP + E2, VCaP ERα + E2 expressing cells and in NCI-H660 cells with and without E2 treatment. Cells were

treated with E2 for 48hrs. ChIP experiments were carried out using Millipore EZ-Magna ChIP kit (Catalogue # 17-10086). Briefly 5-10 X 10^6 cells were crosslinked with 1% formaldehyde for 10 min at room temperature. The crosslinking was then quenched with 0.125 M glycine. Chromatin was sonicated in the lysis buffer to 300-500 bp and the extraction of ChIP DNA was done as per the kit protocol.

For ChIP sequencing the concentrations of the ChIP DNA were quantified by Qubit Fluorometer (Invitrogen). ChIP DNA was prepared into libraries and direct sequencing of the ChIP libraries was performed using illumine Genome Analyzer according to standard manufacturers procedures.

ChIP seq data analysis:

Peak detection for all ChIP-seq experiments was performed with ChIPseeqer[5], using the same parameters for all datasets (i.e., p-value threshold for peaks=10^{-5}, minimum distance between peaks=100bp). Genomic annotation of ChIP-seq peaks, comparison between ChIP-seq datasets and motifs analysis were performed using the corresponding tools in the ChIPseeqer software.

We ranked the binding sites according to their p-value as determined by ChIPSeeqer and considered the expression levels of the potential target genes. In this case, we defined a target gene if it is within 20KB of the peak and considered only genes whose expression is higher than 1 in at least one condition (either VCAP con or VCAP ERα).

Association of NEAT1 co-related signature with Oncomine concepts

Similar to the ERα and NEAT1 signature we also created a NEAT1 gene signature considering the genes that were positively correlated (correlation > 0.5) to NEAT1

expression across prostate cancer datasets in Oncomine and looking for overlapping genes with the ERα 588 gene signature we identified 155 overlapping genes. We defined this NEAT1-ERα signature as an Oncomine custom concept and determined significantly associated tumor vs normal concepts with odds ratio > 3.0 and $P < 1 \times 10^{-6}$. These results are represented as a network using Cytoscape version 2.8.2.

Dataset of long non-coding RNAs (lncRNAs):

We generated a reference set of known ncRNAs from various sources, listed hereafter:

- RefSeq[6]: *6,499 ncRNAs* downloaded from UCSC Table Browser by selecting those without protein coding information, i.e. cdsStart=unk and csdEnd=unk. The dataset was downloaded on 2012.01.31. By selecting those longer than 200nt, we considered 4,637 elements.

- GENCODE v7[7]: We then selected for lincRNA (1,390), snoRNA (1,521), and snRNA (1,943) for a total of 4,854 elements, out of which 1,460 are longer than 200nt. hg19 coordinates converted into hg18 coordinates by liftOver. We removed lncRNAs that were already in the RefSeq data set, by excluding those with at least 25% overlap with a RefSeq lncRNA. This resulted in 859 lncRNAs that are unique to GENCODE.

- lncRNAdb.com[8]: We obtained the coordinates of 73 lncRNAs included in the database (personal communication, Marcel Dinger). We then selected those that were not already identified in the previous steps (less than 25% overlap) and longer than 200nt. This resulted in 30 lncRNAs.

- ncRNA.org: We selected different databases provided via the interface table browser: RNAz F_RNAz_set1_90 (36,155)[9], Jones Eddy (7,218) [ncRNA annotation track for the human genome, version hg16 (July 2003) T.A. Jones, S.R. Eddy ({tajones,eddy}@genetics.wustl.edu)2 March 2004]), RNAPomics (1,051)[10],

sasaki_et_al_150 (153)[11], The names refer to the specific table we used. The total number of elements in this set is: 44,577 of which 6,993 are longer than 200nt. We removed those that were already identified (376), thus the final elements remaining was 6,617.

Cumulatively, the total known ncRNAs from the various databases was 12,143. We classified an lncRNA as intergenic if it did not overlap with any of the transcripts in Ensembl (62,074). According to this definition, about 54% of the lncRNAs (6,850) are intergenic; while the remaining are intronic. To compute the overlap between datasets we used BEDtools[12].

Characterization of the lncRNAs:

We characterized the lncRNAs according to their potential of being regulated by ERα. Moreover, we considered several histone marks to provide evidence of transcription.

Estrogen receptor alpha (ERα) binding:

ChIP-sequencing data on two prostate cell line (VCaP ERα and NCI-H660) were used in order to identify the binding sites of the estrogen receptor. The data was analyzed by ChIPseeker and we identified 3,819 ERα binding sites (ERBS) which were then used to classify the lncRNAs. Specifically, we classified an lncRNA as regulated by ERα if an ERBS lies within 5KB upstream to its transcription start site (TSS). According to this definition, 302 (2.49%) intergenic lncRNAs were regulated by 97 (2.54%) ERBS.

We also looked for Estrogen-Responsive Elements (ERE) motifs within the ERα binding sites of ERα regulated lncRNAs. Specifically, we considered both full ERE (137,531) and half ERE (1,966,283) motifs. The location of ERE motifs were obtained from Carrol et

al[13]. Ninety-two out of 97 (95%) ERα regulated intergenic lncRNA had an ERE motif within the binding sites.

We also considered other transcription binding factors that may act as cofactors in estrogen regulation. Specifically we looked at the Activator Protein 1 (AP-1), which is a regulator of gene expression in response to a variety of stimuli. It is a heterodimer composed by several proteins, among which are c-Jun, c-Fos, and JunD. We thus considered the binding sites of these proteins (from the GM12878 Yale TFBS track in UCSC Genome Browser) and identified which are fully contained in ERα binding sites.

Histone Marks

Similarly to the ERα analysis, we characterized the lncRNAs with respect to active marks (H3K4me3 and H3K36me3), and repressive marks (H3K9me3 and H3K27me3), provided by Chinnaiyan et al.[14]. A window of 10Kb was considered to associate a histone mark to an lncRNA.

RNA extraction, sample preparation and sequencing:

For RNA sequencing analysis frozen tissue was cored (1.5 mm biopsy cores) and RNA extracted using TRIzol Reagent (Invitrogen, CA). Tissues were obtained from radical prostectomy series at the Weill Cornell Medical College. All samples were collected with informed consent of the patients and under an Institutional Review Board approved protocol. The extracted RNA was subjected to DNase treatment using a DNA-free TM Kit (Applied Biosystems/ Ambion, Austin, TX, USA). RNA quality was measured using the RNA 6000 Nano Kit on a Bioanalyzer 2100 (Agilent Technologies, Santa Clara, CA, USA). RNA with RIN (RNA integrity number) ≥ 8 was used for subsequent library preparation. Illumina's sample preparation protocol for paired-end sequencing of mRNA

was used, as previously described[15]. The paired end reads were then aligned to the human genome (hg18) using ELAND/CASAVA, as described previously[15,16].

Differential expression analysis:

We performed pair-wise differential expression analysis on a set of paired-end RNAseq samples (26 benign prostate, 40 prostate adenocarcinoma, and 7 neuroendocrine prostate adenocarcinoma) in order to prioritize the experimental validation. **Supplementary dataset 1** shows the details of the sequencing. Briefly, using the Illumina software suite ELAND/CASAVA, reads were simultaneously mapped to the reference genome NCBI36/hg18 without the minor haplotypes and the minor sequences and a splice junction library based on UCSC knownGenes annotation dataset. Reads mapped to the mitochondrial genome were removed. The library was generated using RSEQtools accordingly to the read size[4]. Gene expression (RPKM) was computed on the composite models based on UCSC knownGenes annotation dataset. Raw RPKM values were then log2-transformed after adding 1, and the resulting dataset was quantile normalized, using the R software package "limma" (http://bioinf.wehi.edu.au/limma).
We performed Wilcoxon test for benign vs. PCa and PCa vs. NEPC on the normalized dataset. The candidates in each list were ranked by p-values, after correction for multiple hypothesis testing[17]. Similarly, we computed the RPKM values for the known lncRNAs and performed the same differential analysis on log2-transformed RPKM+1 values.

VCaP and VCaP ERα sequencing:

Strand Specific sequencing was done for VCaP and VCaP ERα expressing cell lines using deoxy-UTP method for library preparation[18] with some modifications. Briefly, Ribo-Zero rRNA removal kit (RZH1046, Epicentre Biotechnologies, Madison, WI) was used to

remove ribosomal RNA followed by incorporation of deoxy-UTP during second strand cDNA synthesis. Subsequently the uridine-containing strand was destroyed and that enabled identification of the transcript orientation.

Reads from each strand were aligned to the reference genome NCBI36/hg18 without the minor haplotypes and the minor sequences. Reads mapped to the mitochondrial genome were removed. The expression of each gene (UCSC knownGenes) and the lncRNAs identified by our method was computed using RSEQtools[4]. Specifically, we used bgrQuantifier, after generating the bedgraph files from the mapped data. The reference annotation file (UCSC knownGene annotation or the known lncRNA dataset), which includes the coordinates of all the genes from both strands, was first split according to strand information if available. This avoided assigning reads to the wrong gene on the opposite strand when computing the expression levels. The resulting expression data was used to identify variation in gene expression between the control and ERα expressing cells. We computed the ratio between VCaP and VCaP ERα, after adding 1, and selected those genes with a log2-fold change greater than 2. Results are reported in **Supplementary dataset 3**.

Preparation of NEAT1 RNA ISH probe:

The full length NEAT1 gene was cloned in pCRII-TOPO (Invitrogen) vector. The plasmid was digested with NotI (New England Biolabs) and purified with the Qiaquick PCR Purification Kit (Qiagen) according to manufacturer's protocol. In vitro transcription was accomplished using 500ng of linearized plasmid DNA and the MEGAscript SP6 kit (Ambion) as directed by the manufacturer. The resultant RNA was cleaned using the RNeasy kit (Qiagen) using the manufacturer's protocol. 5ug of RNA was mixed with 10ul of buffer A, 5μl of ML DNP reagent (both provided by Ventana-Roche) and water to

50μl. The reaction was incubated at 37°C for two hours. The labeled RNA was cleaned with the RNeasy Kit (Qiagen). 50-250ng/ml of probe was mixed in Ribohybe solution (Ventana-Roche) and 100ul of the probe was used for each slide. RNA in situ hybridization on FFPE slides was performed using an automated protocol developed for the Discovery XT automated staining system (Ventana-Roche).

Supplementary References:

1 Dimple, C. *et al.* Role of PELP1/MNAR signaling in ovarian tumorigenesis. *Cancer Res* **68**, 4902-4909, doi:10.1158/0008-5472.CAN-07-5698 (2008).

2 Nair, S. S. *et al.* PELP1 is a reader of histone H3 methylation that facilitates oestrogen receptor-alpha target gene activation by regulating lysine demethylase 1 specificity. *EMBO Rep* **11**, 438-444, doi:embor201062 [pii] 10.1038/embor.2010.62 (2010).

3 Dobin, A. *et al.* STAR: ultrafast universal RNA-seq aligner. *Bioinformatics* **29**, 15-21, doi:10.1093/bioinformatics/bts635 (2013).

4 Habegger, L. *et al.* RSEQtools: a modular framework to analyze RNA-Seq data using compact, anonymized data summaries. *Bioinformatics* **27**, 281-283, doi:10.1093/bioinformatics/btq643 (2011).

5 Giannopoulou, E. G. & Elemento, O. An integrated ChIP-seq analysis platform with customizable workflows. *BMC bioinformatics* **12**, 277, doi:10.1186/1471-2105-12-277 (2011).

6 Pruitt, K. D., Tatusova, T., Brown, G. R. & Maglott, D. R. NCBI Reference Sequences (RefSeq): current status, new features and genome annotation policy. *Nucleic Acids Res* **40**, D130-135, doi:10.1093/nar/gkr1079 (2012).

7 Harrow, J. *et al.* GENCODE: The reference human genome annotation for The ENCODE Project. *Genome Res* **22**, 1760-1774, doi:10.1101/gr.135350.111 (2012).

8 Amaral, P. P., Clark, M. B., Gascoigne, D. K., Dinger, M. E. & Mattick, J. S. lncRNAdb: a reference database for long noncoding RNAs. *Nucleic Acids Res* **39**, D146-151, doi:10.1093/nar/gkq1138 (2011).

9 Washietl, S., Hofacker, I. L. & Stadler, P. F. Fast and reliable prediction of noncoding RNAs. *Proc Natl Acad Sci U S A* **102**, 2454-2459, doi:10.1073/pnas.0409169102 (2005).

10 Rederstorff, M. *et al.* RNPomics: defining the ncRNA transcriptome by cDNA library generation from ribonucleo-protein particles. *Nucleic Acids Res* **38**, e113, doi:10.1093/nar/gkq057 (2010).

11 Sasaki, Y. T. *et al.* Identification and characterization of human non-coding RNAs with tissue-specific expression. *Biochem Biophys Res Commun* **357**, 991-996, doi:10.1016/j.bbrc.2007.04.034 (2007).

12 Quinlan, A. R. & Hall, I. M. BEDTools: a flexible suite of utilities for comparing genomic features. *Bioinformatics* **26**, 841-842, doi:10.1093/bioinformatics/btq033 (2010).

13 Ross-Innes, C. S. *et al.* Cooperative interaction between retinoic acid receptor-alpha and estrogen receptor in breast cancer. *Genes Dev* **24**, 171-182, doi:10.1101/gad.552910 (2010).

14 Yu, J. *et al.* An integrated network of androgen receptor, polycomb, and TMPRSS2-ERG gene fusions in prostate cancer progression. *Cancer cell* **17**, 443-454, doi:10.1016/j.ccr.2010.03.018 (2010).

15 Pflueger, D. *et al.* Discovery of non-ETS gene fusions in human prostate cancer using next-generation RNA sequencing. *Genome Res* **21**, 56-67, doi:10.1101/gr.110684.110 (2011).

16 Chakravarty, D. *et al.* Extranuclear functions of ER impact invasive migration and metastasis by breast cancer cells. *Cancer Res* **70**, 4092-4101, doi:10.1158/0008-5472.CAN-09-3834 (2010).

17 Benjamini, Y., and Yosef Hochberg. Controlling the False Discovery Rate: A Practical and Powerful Approach to Multiple Testing. *Journal of the Royal Statistical Society. Series B (Methodological)* **57**, 289–300 (1995).

18 Parkhomchuk, D. *et al.* Transcriptome analysis by strand-specific sequencing of complementary DNA. *Nucleic Acids Res* **37**, e123, doi:10.1093/nar/gkp596 (2009).
